# Combined mesenchymal stem cells and metformin therapy modulates key macromolecular pathways in pulmonary fibrosis based on evidence from untargeted metabolomics

**DOI:** 10.1038/s41598-026-46691-8

**Published:** 2026-04-24

**Authors:** Karim Morsi, Taghreed Khaled Abdelmoneim, Nada A. Youssef, Emad M. Elzayat, Sameh Magdeldin, Dina Amr

**Affiliations:** 1 Biotechnology Department, Faculty of Science, Cairo University, Giza, 12613 Egypt; 2 Proteomics and Metabolomics Research Program, Basic Research Unit, Research Department, Children’s Cancer Hospital Egypt , Cairo, 57357 Egypt; 3 Physiology Department, Faculty of Veterinary Medicine, Suez Canal University, Ismailia, Egypt

**Keywords:** Idiopathic pulmonary fibrosis, Mesenchymal stem cells, Metformin, Untargeted metabolomics, LC-MS, Biomarkers, Biochemistry, Cell biology, Diseases, Drug discovery, Medical research, Molecular biology

## Abstract

Idiopathic pulmonary fibrosis (IPF) is a progressive and fatal interstitial lung disease with limited therapeutic options, highlighting the need for novel interventions. Mesenchymal stem cells (MSCs) possess immunomodulatory and regenerative capacities, while metformin, a widely used antidiabetic agent, has recently shown anti-fibrotic potential through metabolic reprogramming. Metabolomic profiling offers a comprehensive approach to elucidate disease-associated biochemical alterations and therapeutic mechanisms. In this pilot study, bleomycin-induced pulmonary fibrosis in rats was treated with MSCs, metformin, or their combination. Untargeted LC-MS-based metabolomics of plasma and lung tissue, alongside histopathological evaluation, revealed that combination therapy most effectively corrected metabolic disruptions and attenuated fibrotic remodeling. Histology confirmed marked reductions in alveolar wall thickening, collagen deposition, and fibroblastic foci in the combination group compared to monotherapies. Metabolite changes in lung tissue included sphingosine (lipid metabolism), 5-hydroxyindoleacetic acid (serotonin turnover), cyclic GMP (nitric oxide signaling), and 4-guanidinobutanoic acid (arginine/creatine metabolism). Plasma regulation involved corticosterone (steroid biosynthesis), PC (18:2/18:2) (phospholipid remodeling), methionine (methylation balance), galactose 1-phosphate (carbohydrate metabolism), and niacinamide (NAD⁺ biosynthesis). These findings provide preliminary evidence that combined MSCs–metformin therapy synergistically ameliorates fibrosis and metabolic dysregulation, while identifying metabolites that may serve as potential biomarkers for disease progression and therapeutic response.

**Supplementary Information:**

The online version contains supplementary material available at 10.1038/s41598-026-46691-8.

## Introduction

Idiopathic pulmonary fibrosis (IPF) is a devastating chronic interstitial lung disease of unknown etiology, primarily affecting older adults. It is characterized by progressive scarring of lung parenchyma, leading to impaired gas exchange and respiratory failure. Median survival is approximately 3–5 years after diagnosis despite currently available therapies^1^. Histologically, IPF is associated with the usual interstitial pneumonia (UIP) pattern, marked by fibroblastic foci, extracellular matrix accumulation, and loss of alveolar integrity^2^. The disease pathogenesis involves a complex interplay between repetitive epithelial injury, aberrant wound healing, fibroblast proliferation, and myofibroblast differentiation, all contributing to excessive collagen deposition and architectural distortion^3^.

Metabolic reprogramming is increasingly recognized as a determinant of fibrotic progression in IPF. Injured alveolar epithelial cells demonstrate mitochondrial dysfunction, endoplasmic reticulum stress, and perturbed lipid handling, thereby fostering a pro-fibrotic milieu^4^. Consistent alterations across clinical and experimental studies implicate dysregulation of amino-acid metabolism, fatty-acid oxidation, glycolysis, and nucleotide biosynthesis^5,6^. In particular, enhanced glycolytic flux with concomitant suppression of oxidative phosphorylation in lung fibroblasts has been linked to myofibroblast activation and collagen production^7^. Moreover, specific metabolites—including corticosterone, PC(18:2(9Z,12Z)/18:2(9Z,12Z)), methionine, galactose-1-phosphate, niacinamide, sphingosine, 5-hydroxyindoleacetic acid (5-HIAA), cyclic GMP, and 4-guanidinobutanoic acid—have been reported as candidate biomarkers or mediators of fibrotic signaling, underscoring a substantial metabolic shift during disease initiation and progression^8^.

The pathogenesis of IPF also involves the dysregulation of immune and inflammatory responses. A key upstream event is the dysfunction of alveolar epithelial type II cells, which triggers the release of pro-fibrotic cytokines such as transforming growth factor-beta (TGF-β), interleukin-6 (IL-6), and connective tissue growth factor (CTGF), initiating fibroblast proliferation and extracellular matrix deposition^9^. Notably, TGF-β1 is a master regulator of fibrosis, driving epithelial-to-mesenchymal transition, fibroblast activation, and suppression of lipogenic pathways. This signaling cascade is tightly coupled with cellular metabolism, including AMP-activated protein kinase (AMPK) inhibition and increased reactive oxygen species (ROS) generation, further amplifying fibrogenesis^10^.

The use of metabolomics, particularly liquid chromatography–mass spectrometry (LC-MS/MS)-based untargeted profiling, has enabled comprehensive characterization of the metabolic milieu in IPF. This approach allows the identification of systemic metabolic alterations and their modulation in response to therapeutic interventions. Studies have shown significant changes in amino acid levels, bile acid composition, lipid intermediates, and purine metabolism in IPF models, suggesting that metabolomics can serve both diagnostic and prognostic roles^11,12^.

Given the limitations of current antifibrotic agents such as nintedanib and pirfenidone—both of which only slow disease progression—there is an urgent need for novel therapeutic strategies that address the metabolic and cellular basis of IPF^13,14^. Mesenchymal stem cells (MSCs), derived from bone marrow or adipose tissue, represent a promising cell-based therapy owing to their immunomodulatory, anti-inflammatory, and regenerative capabilities. These cells can home to injured lung tissue, suppress TGF-β1 signaling, and restore alveolar structure by promoting epithelial repair^15^. In addition, MSCs-derived extracellular vesicles carry antifibrotic microRNAs and proteins that modulate the fibrotic microenvironment, offering a cell-free adjunct or alternative to direct MSCs administration^16^.

Alongside these developments, metformin—a biguanide class oral hypoglycemic agent—has emerged as a potential antifibrotic agent beyond its metabolic role in diabetes. Metformin activates AMPK, a central energy sensor, which inhibits TGF-β1-induced fibrotic signaling, suppresses myofibroblast activation, and promotes lipogenic reprogramming in fibroblasts^17^. Preclinical studies have demonstrated that metformin attenuates bleomycin-induced pulmonary fibrosis, reduces collagen deposition, and improves lung compliance^18^. Furthermore, its favorable safety profile and low cost make it an attractive candidate for repurposing in fibrotic lung diseases.

Because, mesenchymal stem cells (MSCs) primarily modulate immune dysregulation, suppress inflammation, and promote tissue repair, whereas metformin exerts anti-fibrotic effects through metabolic reprogramming and inhibition of pro-fibrotic signaling pathways^16,18^. Therefore, their combination may provide complementary and synergistic therapeutic benefits in idiopathic pulmonary fibrosis. Given the multifactorial nature of IPF pathogenesis, targeting both cellular and metabolic drivers of fibrosis may achieve greater efficacy than either strategy alone.

In light of these considerations, using integrated untargeted LC–MS/MS metabolomics and histopathological analyses to evaluate whether combined MSCs and metformin therapy more effectively restores metabolic homeostasis and attenuates fibrotic remodeling than either monotherapy in a bleomycin-induced rat model of pulmonary fibrosis. In addition, we aimed to identify potential metabolic biomarkers associated with therapeutic response that may inform future translational and clinical studies.

## Materials and methods

### Materials

#### Animal models

Thirty-five adult male albino Wistar rats, weighing 150–200 g, were utilized in this study.

All animals underwent histopathological assessment. For metabolomics, due to sample availability, 17 plasma samples and 15 lung-tissue samples were analyzed.

#### Reagents

Bleomycin was employed for the induction of IPF. Therapeutic agents comprised mesenchymal stem cells (adipocyte-derived) and metformin.

### Methods

#### Experimental design

**Fig. 1:**
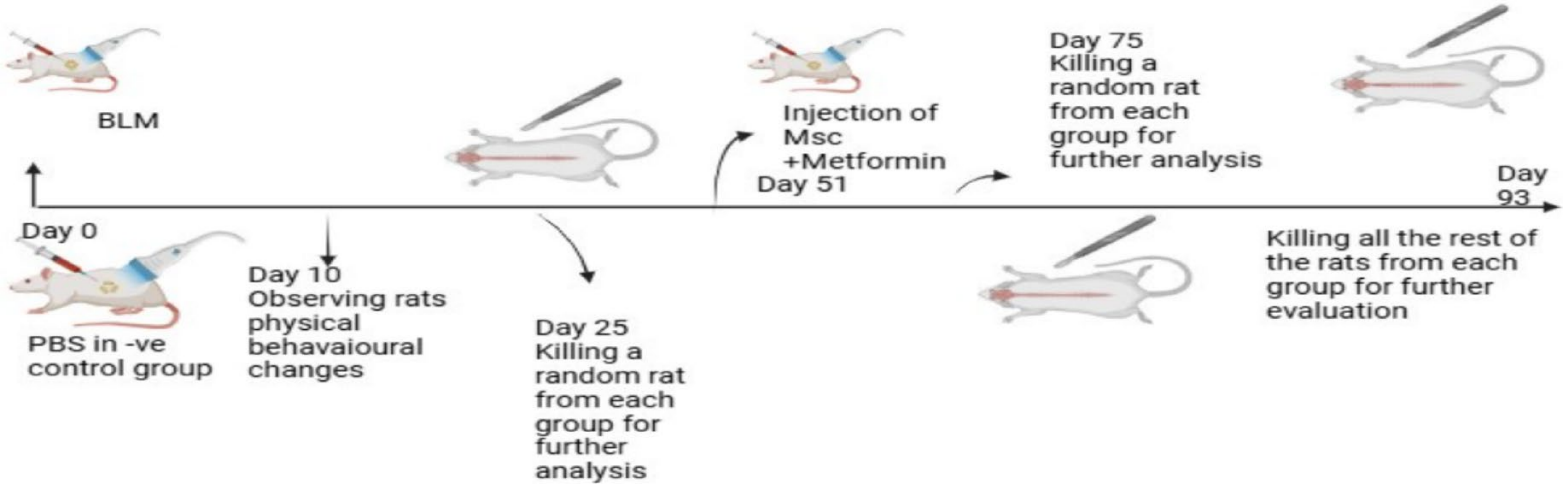
Schematic representation of the expremiental design. Where, IPF was induced by a single intratracheal (IT) instillation of bleomycin (BLM) at a dose of 5 mg/kg. Metformin was administered orally at a dose of 65 mg/kg. Mesenchymal stem cells (MSCs) were administered as a single intravenous (IV) injection via the tail vein at a concentration of 1 × 10⁶ cells per rat.

This study consisted of two primary phases: an induction phase and a therapeutic phase (Fig. 1).

Induction Phase (51 days) Pulmonary fibrosis was induced using bleomycin. Prior to induction, baseline serum samples were collected from 10 rats and stored at −80 °C for subsequent metabolomics analysis (Control Group reference).

Therapeutic Phase (42 days): Following the induction phase, rats were allocated to treatment groups. Treatments included metformin (65 mg/kg body weight, administered orally every 48 h), MSCs (a single dose of 1 million cells), or a combination of both. Serum samples were collected post-treatment for metabolomics analysis.

#### Verification of IPF induction

Histopathological examination of lung tissue was conducted to confirm the successful induction of IPF. Collagen deposition, a key indicator of fibrosis, was assessed, potentially using methods like hydroxyproline quantification^5^.

#### Sample preparation for metabolomics analysis

The plasma and tissue metabolites extraction protocol is carried out through a series of precuse steps to ensure high quality sample preparation for LC-MS analysis, using MS-DIAL (version 4.9.2; https://computationalmetabolomics.osaka-u.ac.jp/cms/software/msdial). Additional spectral analysis and compound identification were conducted using PeakView Software (version 2.2; https://sciex.com/products/software/peakview-software) and MasterView Software (version 1.1; https://sciex.com/products/software/masterview-software), the corresponding figure is presented in the Supplementary Materials (Supplementary Figure S1). The procedure used to prepare the samples was based on earlier research^19,21,21,22^. Lung tissue samples were mixed with a pre-cooled protein precipitation buffer (chloroform: methanol: MilliQ water, 1:3:1) in a 1:8 ratio and homogenized. For plasma samples, the procedure begins with thawing the plasma sample and vortexing it for a few seconds to ensure homogeneity. Then, the plasma were mixed with the protein precipitation buffer in a 1:8 ratio and homogenized.

For both plasma and tissue, the mixture was sonicated (Elma Elmasonic Ultrasonic cleaning unit P, Elma, Germany) for 20 min at 80 kHz at 100% power (at ambient temperature). The supernatant was collected, evaporated using a speed vacuum (Concentrator plus Eppendorf, Germany) for three hours (at ambient temperature), and then re-suspended in reconstitution buffer (H2O: methanol: ACN, 2:1:1) at a volume corresponding to the starting tissue weight followed by centrifugation (10,000 rpm, 10 min, 4 °C) (Tomy MX-207 High Speed Refrigerated Micro Centrifuge, Tomy Kogyo Co., Ltd, Japan). For later LC-MS analysis, samples were moved to LC vials^22^.

#### UHPLC-MS/MS analysis

The samples were analyzed using LC-MS/MS in both positive and negative ionization modes using Information Dependent Acquisition (IDA)^23^. The overall UHPLC–MS/MS analytical workflow is illustrated in Supplementary Figure S2 .The UHPLC-MS/MS analysis was performed using an ExionLCTM AC UHPLC system (AB SCIEX, Concord, Canada) with an Acquity XSelect HSS T3 analytical column 2.1 × 150 mm, 2.5 μm (Waters Co, Milford, US) in conjunction with a Triple TOF 5600 + mass spectrometer (AB SCIEX, Concord, Canada).

For chromatographic separation, 10µL of each sample was injected and run for 35 min with a constant flow rate of 0.3 mL/min using gradient elution. The mobile phases consisted of: (A) 5 mM ammonium formate in 1% methanol (pH 3.0) for positive mode; (B) 5 mM ammonium formate in 1% methanol (pH 8.0) for negative mode; and (C) 100% acetonitrile. The gradient elution program was: 0% (C) for 1 min, a linear gradient of 0–90% (C) over 20 min, 90% (C) for 4 min, a linear gradient of 90 − 0% (C) over 1 min, and finally, a 3-minute re-equilibration at 0% (C)^24,25^. Mass spectrometry was performed in both positive (ESI+) and negative (ESI-) ion modes using a DuoSpray ion source.

To detect metabolites within the samples, IDA acquisition with dynamic background subtraction was employed. A TOF-MS scan from 50 to 1000 Da was performed in 30 ms, followed by MS/MS on the 15 most intense precursor ions from 50 to 1000 Da using a fixed 50 Da transition window. The MS/MS acquisition time was 50 ms, with collision energies of 35 V and − 35 V for positive and negative modes, respectively. The whole cycle time was 0.6502 s. Analyst TF (v 1.7.1) was used to collect MS and MS/MS spectra^23^.

Pooled samples were generated and used as the study reference and for quality control (QC) checks. Besides, an automated calibration delivery system performed mass calibration every 2 h using either positive or negative APCI calibration solution (AB SCIEX). Blank specimen was employed after each sample to clean up any possible carry over and to assess the quality of all runs^25,26^.

Pooled samples were generated and used as the study reference and for quality control (QC) checks. Besides, an automated calibration delivery system performed mass calibration every 2 h using either positive or negative APCI calibration solution (AB SCIEX). Blank specimen was employed after each sample to clean up any possible carry over and to assess the quality of all runs^25,26^.

### Statistical analysis and data pre-processing

Data pre-processing, statistical analyses, and visualization were performed using R (version 4.2.2)^27^. Metabolite abundance data, compiled into a single file (“Gnp table”) using MS-DIAL, underwent several pre-processing steps. Probabilistic quotient normalization (PQN) was applied to all samples before filtering^28^. Metabolite features with missing values in any sample within a group were removed. Duplicate features were filtered, retaining the highest intensity feature. Missing values were imputed by randomly selecting a value within ± 10% of the group’s median^29^. Auto-scaling was performed before statistical analysis^30^. Shared metabolites were tested for normality using the Shapiro-Wilk test (*p* ≤ 0.05)^31^. Differentially expressed metabolites (DEMs) were identified using the ANOVA test, with additional cutoffs of an adjusted p-value (FDR) ≤ 0.05^32^. Multivariate statistical analysis included principal component analysis (PCA), partial least squares discriminant analysis (PLS-DA), and hierarchical clustering analysis (HCA).

### Bioinformatics analysis

#### Database construction for metabolite identification

A custom, high-resolution spectral database derived from the Human Metabolome Database (HMDB) was created, focusing specifically on blood metabolites to facilitate untargeted metabolomics analysis. This involved developing in-house machine learning algorithms (R scripts, unpublished) for spectral data retrieval and validation. Key refinements included: (1) restriction to experimental LC-MS data, (2) selection of specific experimental adduct types, and (3) inclusion of only endogenous blood metabolites. This curated database enabled a more targeted analysis of blood-related metabolic alterations.

#### Metabolite annotation

Metabolite identification utilized MS-DIAL 4.9.2 and the custom in-house database^26^. Manual validation using Peak View 2.2 with Master View 1.1 (AB SCIEX) increased confidence in annotations at both precursor and fragment ion levels^27^. Annotation criteria included a precursor ion extracted ion chromatogram (XIC) signal-to-noise ratio > 10, a sample-to-blank intensity ratio > 5, and a precursor mass tolerance of 10 ppm. Annotated data were subsequently used for pre-processing and downstream statistical analyses^23^.

#### KEGG pathway mapping

To delineate metabolic pathways impacted by IPF and treatment interventions, bioinformatics analysis was performed using the Kyoto Encyclopedia of Genes and Genomes (KEGG)^33^. Significantly altered metabolites, identified via MetaboAnalyst 5.0, were mapped to corresponding KEGG metabolic pathways. Pathway enrichment analysis assessed the effects of different treatments (IPF+Metformin, IPF+MSCs, IPF+MSCs+Metformin), using a Benjamini-Hochberg adjusted p-value threshold of 0.05. KEGG Mapper facilitated the generation of integrated pathway maps and the identification of functional interactions^34^. Pathway associations were further cross-validated using the Reactome database^35^ to ensure the functional coherence of identified metabolic networks.

#### Animal procedures and anathesia

No general anesthesia was administered since the study did not involve surgical interventions. To minimize discomfort associated with experimental handling, rats received the opioid analgesic keto-morphine at a dose of 2 mg/kg orally once daily during the procedure period. Animals were observed twice daily using a standardized pain/distress checklist, and no signs of excessive sedation or distress were noted. This approach ensured effective analgesia while avoiding unnecessary anesthesia exposure.

#### Ethics statement

All experimental protocols were approved by the Institutional Animal Care and Use Committee of [Cairo University], approval number [0000715]. All procedures involving animals were carried out in accordance with relevant national and institutional guidelines and regulations. Reporting of animal experiments in this study complies with the ARRIVE guidelines (https://arriveguidelines.org).

## Results

### Study subjects

In this experimental study, a total of thirty-five male Wistar rats (weighing 150–200 g) were enrolled and randomly assigned to five cohorts. The animals were randomly allocated into five cohorts: Healthy Control (*n* = 7), IPF (*n* = 7), Metformin-treated (*n* = 7), Mesenchymal Stem Cell (MSCs)-treated (*n* = 7), and Combined Metformin + MSCs-treated (*n* = 7).

All animals underwent histopathological assessment. For metabolomics, due to sample availability, 17 plasma samples and 15 lung-tissue samples were analyzed. IPF was induced with bleomycin in all groups except Control, while treatment groups received metformin, MSCs, or their combination as detailed in Methods section. Healthy Controls received vehicle only.

Body weight changes are presented in the Supplementary Materials (Supplementary Table S1 and Supplementary Fig. S3 ).

### Metabolite annotation

Plasma and lung tissue samples were collected from all experimental cohorts (Healthy control, IPF, Metformin-treated, Mesenchymal-treated, and Combined MSCs+Metformin-treated) for untargeted metabolomic profiling. Analyses were performed using liquid chromatography–tandem mass spectrometry (LC–MS/MS) in both positive and negative electrospray ionization (ESI) modes. The initial screening generated 484 features in positive mode and 701 features in negative mode. Following preprocessing—peak detection/alignment (MS-DIAL 4.9.2), PQN normalization, duplicate/adduct consolidation, a ± 10 ppm mass-error cutoff, and manual curation—a refined dataset was generated for both plasma and tissue samples. In plasma, 72 annotated metabolites were detected across all five cohorts (relatively quantified), whereas 40 annotated metabolites were cohort-restricted (absent or below threshold in ≥ 1 cohort). In lung tissue, 117 annotated metabolites were shared across all cohorts, and 20 were cohort-restricted (Supplementary Table 2, Excel File 1 (.csv).).

### Metabolite classification by main classes

**Fig. 2:**
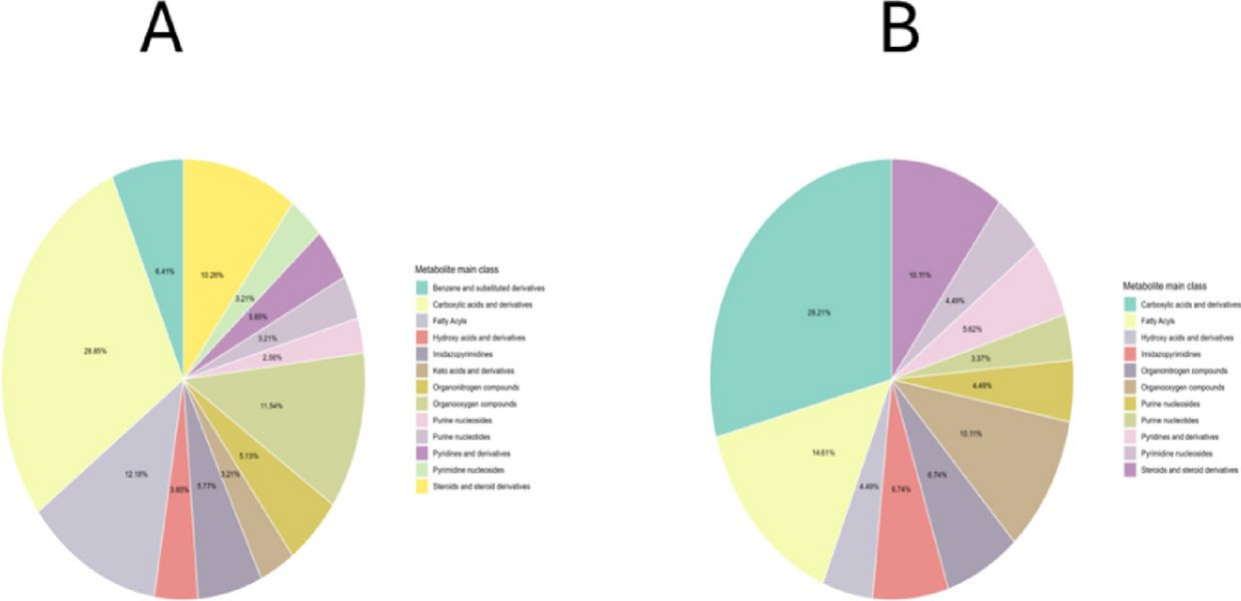
Distribution of identified metabolites by main chemical classes in plasma and tissue. (**A**) Plasma metabolites, with carboxylic acids and derivatives (28.85%) and lipid-like molecules (25.96%) representing the largest proportions. (**B**) Tissue metabolites, with carboxylic acids and derivatives (29.21%) and lipids and lipid-like molecules (25.84%) as dominant classes. Percentages indicate relative abundance of each metabolite class.

To provide an overview of the biochemical landscape, metabolites were categorized according to their main chemical classes. Plasma and tissue metabolomes showed broadly similar profiles, dominated by carboxylic acids and lipids, with minor contributions from other classes (Fig. 2).

These distributions indicate that amino acid–derived and lipid-related metabolites constitute the core of both systemic and tissue-level metabolic remodeling in IPF, providing a biochemical basis for subsequent pathway and univariate analyses.

### Histopathological findings

**Fig. 3:**
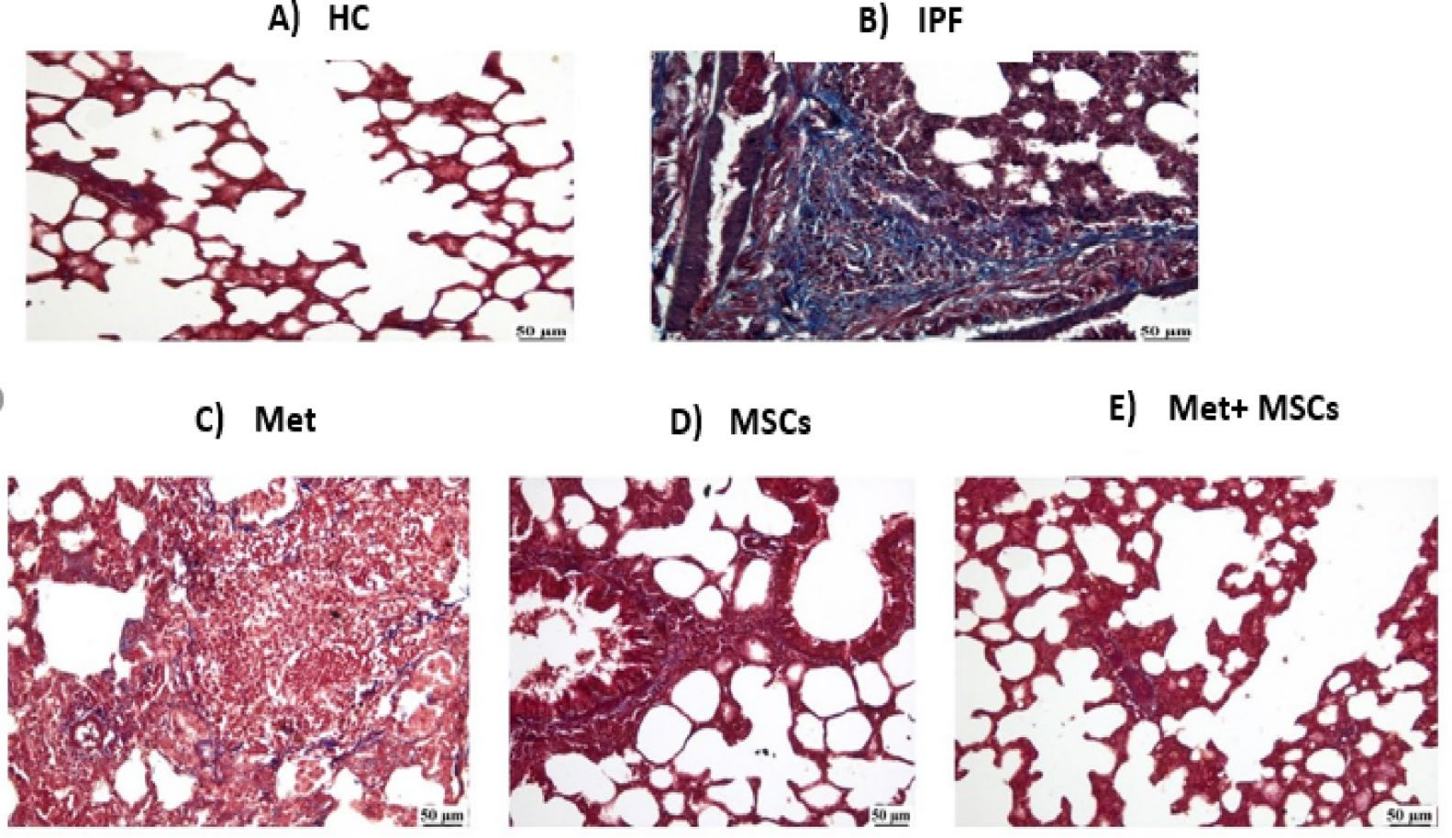
Masson’s trichrome–stained lung sections (collagen, blue; parenchyma, red). (**A**) Healthy Control (HC); (**B**) Idiopathic pulmonary fibrosis (IPF); (**C**)Metformin-treated (Met); (**D**) Mesenchymal Stem Cell (MSCs)-treated; (**E**) Combined Metformin + MSCs-treated. Scale bar, 50 μm.

Masson’s trichrome staining revealed distinct, group-dependent remodeling of the lung parenchyma (Fig. 3). Healthy Control animals displayed normal alveolar architecture with minimal interstitial collagen. In contrast, the IPF group exhibited diffuse collagen deposition, thickened alveolar septa, and architectural distortion consistent with advanced fibrosis. The Metformin-treated group showed fewer fibrotic foci and partial preservation of alveolar spaces relative to IPF. The Mesenchymal Stem Cell (MSCs)-treated group demonstrated moderate fibrosis with improved alveolar spacing, comparable to metformin monotherapy. The Combined Metformin + MSCs-treated group showed the greatest attenuation of fibrosis, with near-normal architecture and minimal collagen. Quantitatively, fibrosis area did not differ between the Metformin-treated and MSCs-treated groups, whereas the Combined Metformin + MSCs-treated group exhibited a significant reduction versus each monotherapy and versus IPF.

### Univariate statistical analysis

**Fig. 4:**
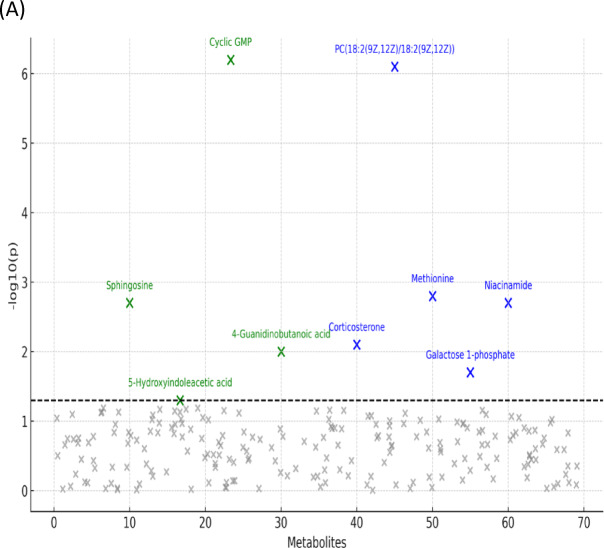

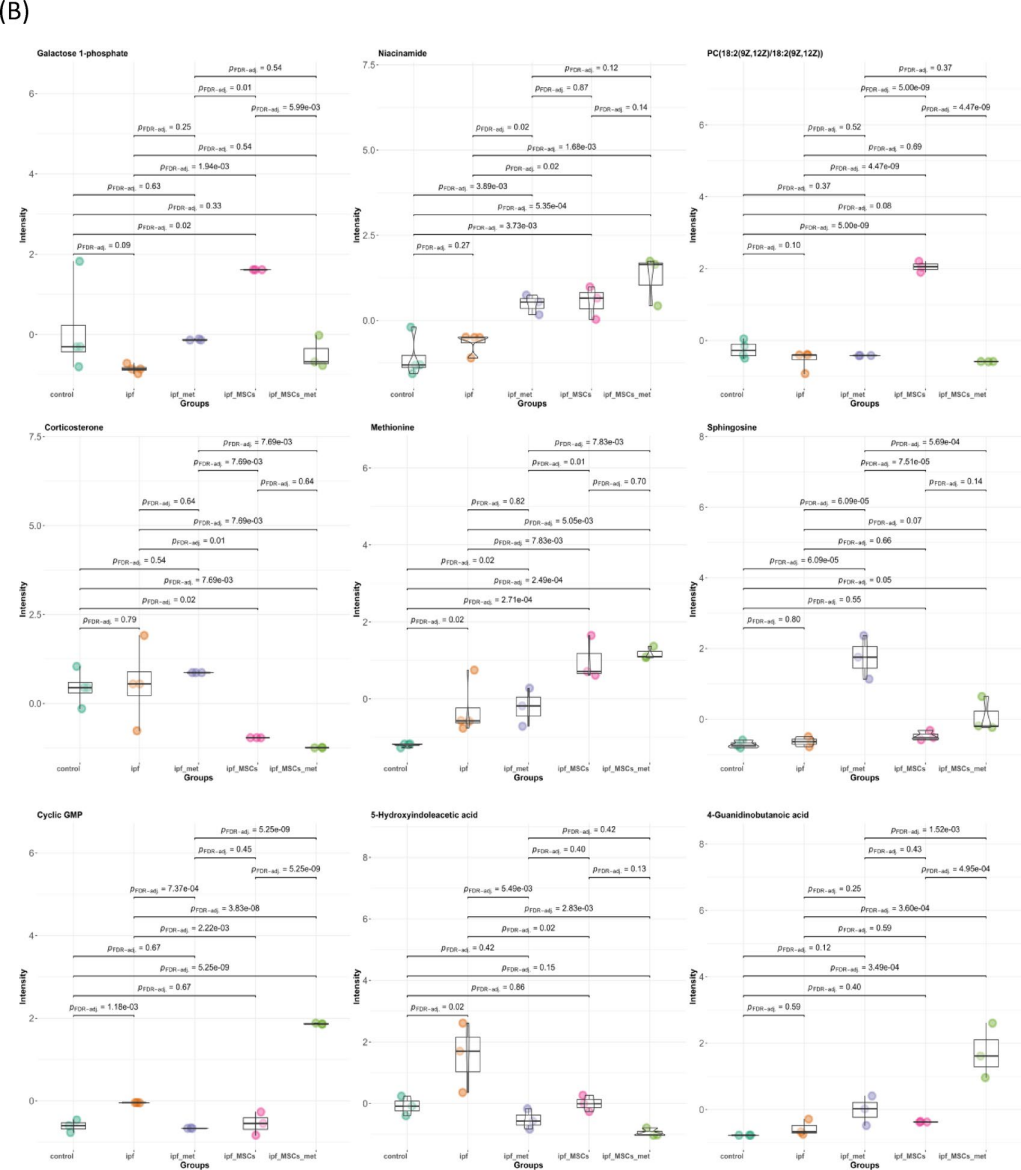
Univariate analysis of plasma and lung-tissue metabolomics profiles. (**A**) Scatter plot of −log10 (p) for all tested metabolites. The nine significant DEMs (FDR ≤ 0.05, Benjamini–Hochberg) are highlighted and labeled; blue asterisks denote plasma metabolites and green asterisks denote tissue metabolites. The dashed horizontal line indicates the nominal significance threshold (p = 0.05; y = −log10 (0.05) =1.3).(**B**) Boxplots for the nine differentially expressed metabolites (DEMs) comparing auto scaled intensities across the five cohorts (Healthy Control, IPF, Metformin-treated, MSC-treated, Combined Metformin+ MSCs- treated).

Univariate analyses were undertaken to identify differentially expressed metabolites (DEMs) among the study groups. Prior to hypothesis testing, metabolite intensities were auto scaled (mean-centered and scaled to unit variance). For each metabolite, a one-way analysis of variance (ANOVA) was performed across the five groups, followed—where the omnibus test was significant—by post-hoc pairwise comparisons. P-values were adjusted for multiple testing using the Benjamini–Hochberg false discovery rate (FDR); statistical significance was defined as FDR-adjusted *p* ≤ 0.05. This analysis identified total of 29 metabolites in both plasma and lung tissue with significant differential abundance among the groups, these metabolites were subsequently filtered down to nine metabolites based on biological relevance to IPF pathogenesis (As shown in Fig. 4). Galactose-1-phosphate (plasma; galactose metabolism, map00052) was lower in IPF than in all treatment arms (Metformin-treated, MSC-treated, and Combined), indicating restoration of carbohydrate flux with intervention. Methionine (plasma; one-carbon/cysteine–methionine metabolism, map00270) was higher in IPF than in Healthy Control, MSC-treated, and Combined groups, with no difference between IPF and Metformin-treated, suggesting correction primarily with MSC-containing therapy. Niacinamide (plasma; nicotinate and nicotinamide metabolism, map00760) was higher in IPF than in Metformin-treated and Combined groups but not different from MSC-treated, consistent with treatment-specific effects on NAD⁺ salvage. Corticosterone (plasma; steroid hormone biosynthesis, map00140) was elevated in IPF relative to MSC-treated and Combined, while not differing from Metformin-treated, indicating broader suppression of glucocorticoid tone with MSC-containing regimens.

The phospholipid PC (18:2/18:2) (plasma; glycerophospholipid metabolism, map00564) was higher in IPF than in MSC-treated but not different from Metformin-treated or Combined, implicating an MSC-specific correction of membrane lipid remodeling. Sphingosine (lung tissue; sphingolipid metabolism, map00600) was altered in Metformin-treated versus both Healthy Control and IPF, whereas MSC-treated and Combined showed no difference from IPF, pointing to a Metformin-specific shift in sphingolipid signaling. 5-Hydroxyindoleacetic acid (5-HIAA) (lung tissue; tryptophan/serotonin metabolism, map00380) was higher in MSC-treated than in IPF and Healthy Control, suggesting MSC-mediated modulation of serotonin turnover. Cyclic GMP (cGMP) (lung tissue; cGMP–PKG signaling, map04022) was higher in MSC-treated and Combined compared with IPF, with no difference for Metformin-treated, highlighting MSC-driven restoration of nucleotide signaling. Finally, 4-guanidinobutanoic acid (lung tissue; arginine/proline and urea-cycle–related metabolism, map00330/map00260) was higher in Combined versus IPF, not differing in Metformin-treated or MSC-treated, indicating a distinct metabolic effect of the combination regimen. (All pairwise differences refer to FDR-adjusted comparisons; full statistics are visualized in Fig. 4, summarized in Supplementary Table S2).

In addition to the nine significantly altered metabolites visualized in box plots, two metabolites were identified with group-specific significance. Dihydroxyacetone phosphate (DHAP) was significantly elevated in the IPF group compared to controls (p value = 0.0128), while fructose 1, 6-bisphosphate (F1, 6BP) was significantly decreased in IPF relative to both control (p value = 0.0311) and MSC-treated groups (p value = 0.0087). These metabolites, central intermediates of glycolysis, are presented in as Excel File 1 (.csv).

Collectively, the DEMs span carbohydrate, one-carbon/amino-acid, glucocorticoid, glycerophospholipid, sphingolipid, serotonin, nucleotide-signaling, and nitrogen/urea-cycle pathways. Plasma-restricted signals (e.g., galactose-1-phosphate, methionine, niacinamide, corticosterone, PC (18:2/18:2)) highlight systemic metabolic remodeling, whereas tissue-restricted signals (e.g., sphingosine, 5-HIAA, cGMP, 4-guanidinobutanoic acid) capture local lung biology and treatment effects. The Combined regimen demonstrated the broadest normalization across these axes, suggesting potential additivity or synergy beyond either monotherapy.

**Fig. 5:**
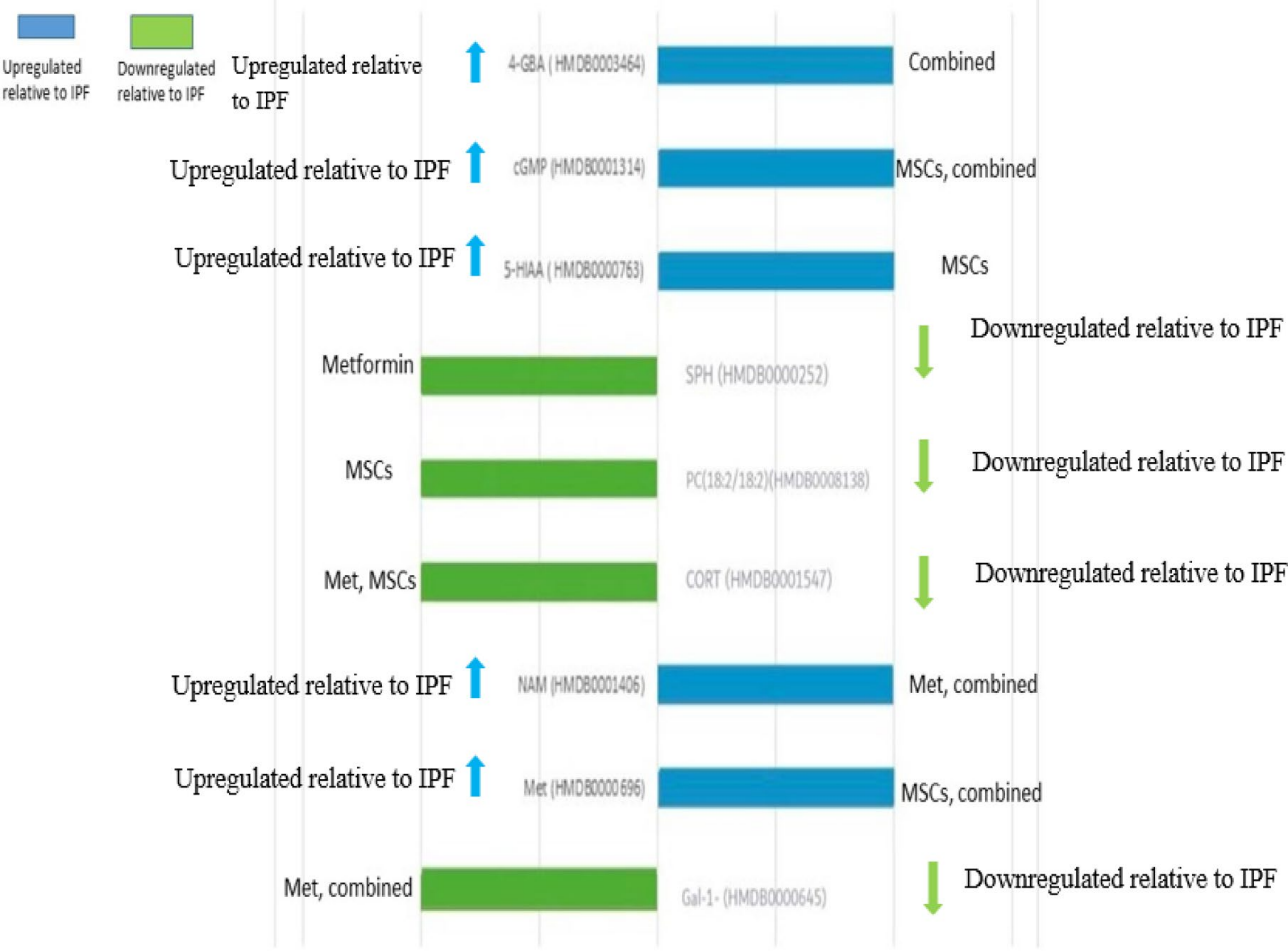
Significantly differentially expressed metabolites (DEMs) identified between idiopathic pulmonary fibrosis (IPF) and treatment groups (Metformin, MSCs, and Combined therapy), as well as compared to Control.

Univariate statistical analysis revealed distinct metabolite alterations between experimental groups; these findings, including relative quantification and adjusted p-values, are illustrated in Fig. 5.

**Fig. 6:**
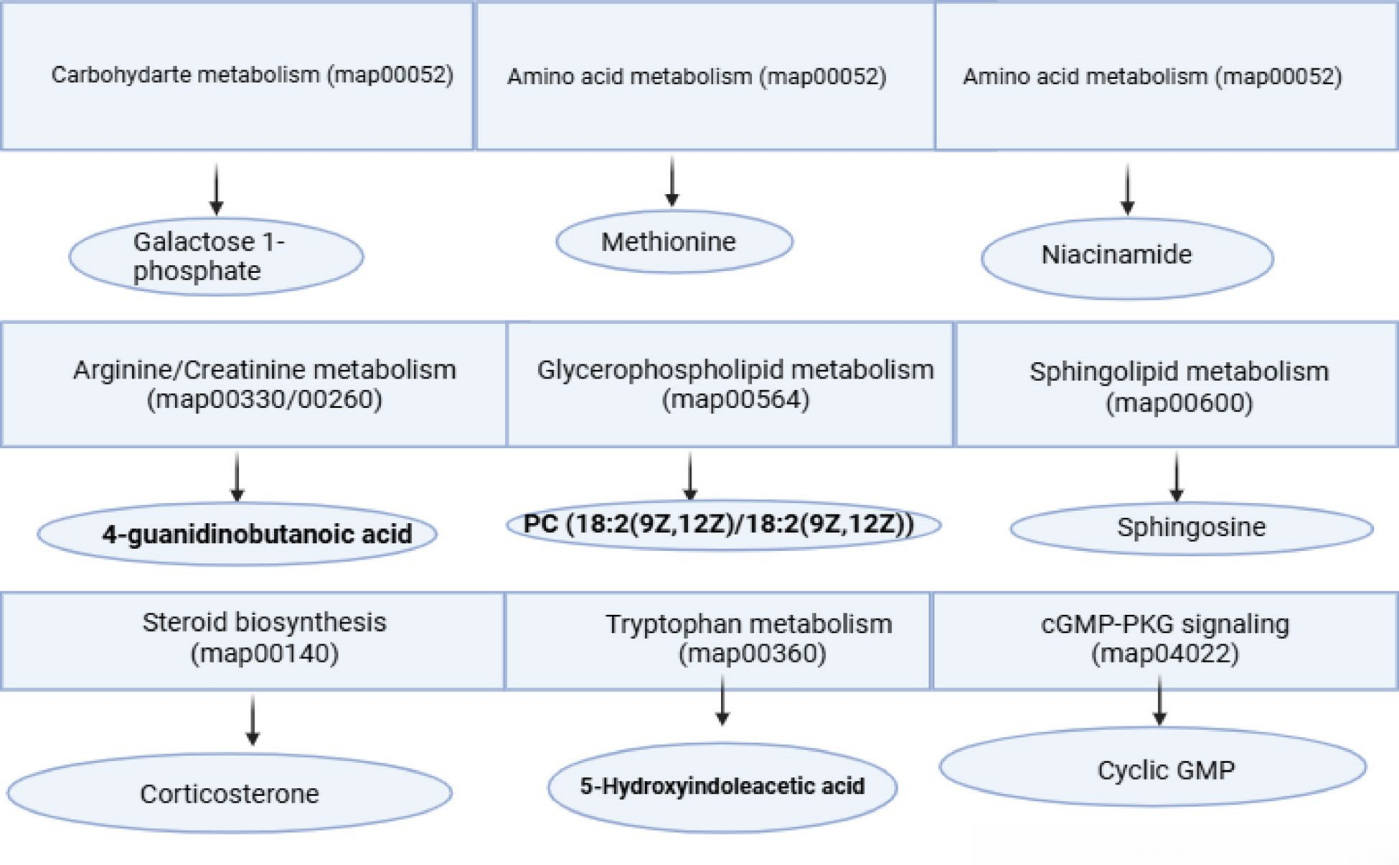
Illustration showing a visual summary of the metabolic pathways and their associated key metabolites. Pathway maps were obtained from the Kyoto Encyclopedia of Genes and Genomes (KEGG; Kanehisa & Goto, 2000; https://www.kegg.jp/). Metabolites (ellipses) are mapped to their primary KEGG pathways (boxes): galactose-1-phosphate—carbohydrate/galactose metabolism (map00052); methionine—amino-acid/one-carbon (map00270); niacinamide—nicotinamide/NAD⁺ (map00760); 4-guanidinobutanoic acid—arginine/creatine & urea-cycle (map00330/map00260); PC(18:2/18:2)—glycerophospholipid (map00564); sphingosine—sphingolipid (map00600); corticosterone—steroid biosynthesis (map00140); 5-HIAA—tryptophan/serotonin (map00360); cGMP—cGMP–PKG signaling (map04022).

To contextualize the metabolomics findings, the significantly altered metabolites were mapped to their corresponding biochemical pathways as shown in Fig. 6.

### Multivariate analysis of plasma and tissue metabolomics profiles

**Fig. 7:**
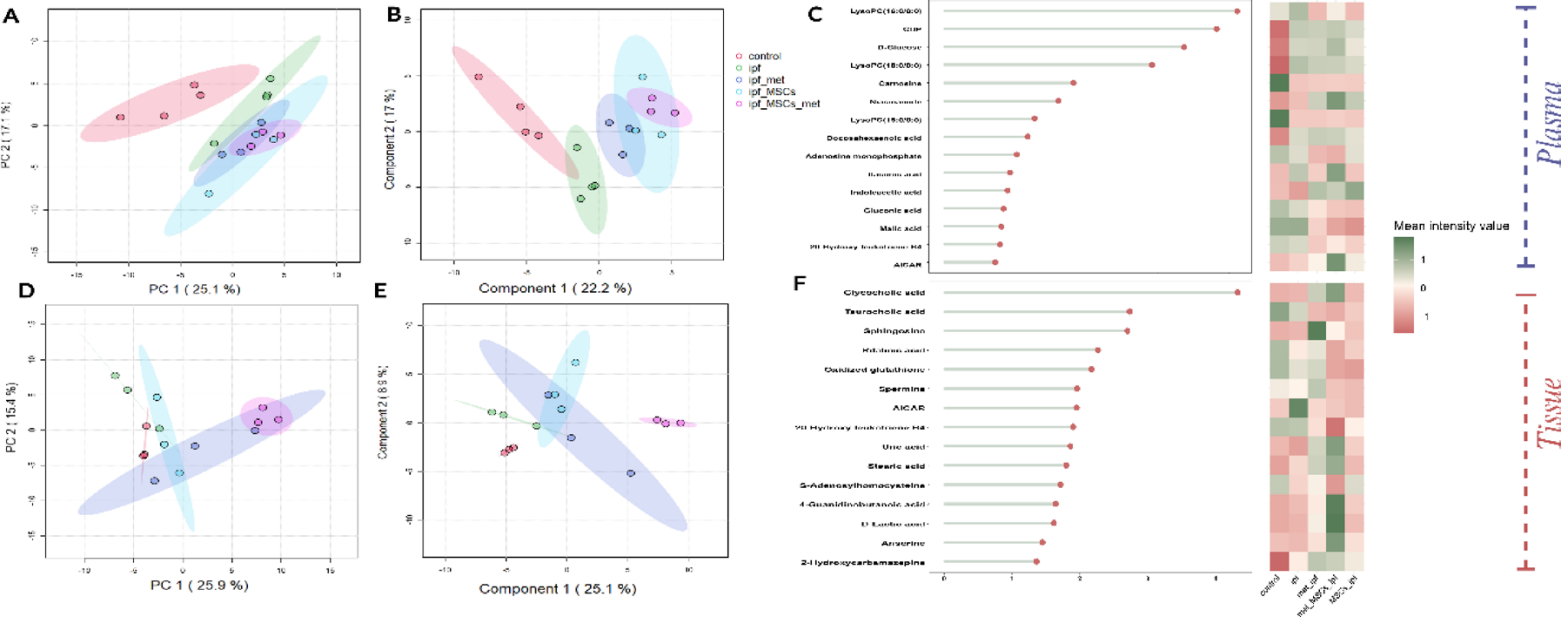
Multivariate analysis of plasma and tissue metabolomics profiles across experimental groups. (**A**) PCA scores plot for plasma. (**B**) PLS-DA scores plot for plasma. (**C**) VIP score plot for plasma highlighting the 9 most important discriminatory metabolites (VIP ≥ 1). (**D**) PCA scores plot for tissue. (**E**) PLS-DA scores plot for tissue. (**F**) VIP score plot for tissue highlighting the 9 most important discriminatory metabolites (VIP ≥ 1).

Unsupervised principal component analysis (PCA) was first used to summarize overall variance and visualize clustering among the five cohorts (Healthy Control, IPF, Metformin-treated, MSC-treated, and Combined Metformin+MSCs-treated). In plasma (Fig. 7A), PC1 and PC2 explained 25.1% and 17.1% of the variance, respectively, and revealed clear separation of IPF from Healthy Control, whereas the treated groups partially overlapped with controls, indicating a shift of the plasma metabolome toward normalization. In lung tissue (Fig. 7D), PC1 accounted for 25.9% and PC2 for 16.4% of the variance, again showing strong discrimination of IPF versus Healthy Control; treatment groups moved toward the control cluster, with the Combined regimen showing the largest shift.

To refine group separation, partial least squares–discriminant analysis (PLS-DA) was applied. In plasma (Fig. 7B), component 1 explained 22.2% and component 2 17.1% of the variance, yielding improved discrimination relative to PCA and clustering of treated groups nearer to controls. In lung tissue (Fig. 7E), component 1 explained 25.1% and component 2 18.9%, producing distinct clustering across groups; the Combined group showed the most pronounced shift toward the control profile. Model validation supported the stability of these patterns (Methods/Supplement). Variable-importance-in-projection (VIP) analyses then identified metabolites most responsible for class separation: in plasma (Fig. 7 C), nine discriminatory metabolites (VIP ≥ 1) were prioritized, including uridine, malic acid, argininosuccinic acid, corticosterone, and lysophosphatidylcholines; in lung tissue (Fig. 7 F), nine priority metabolites included cyclic GMP, sarcosine, xanthine, serine, and 5-hydroxyindoleacetic acid (5-HIAA). Overall, the multivariate results corroborate the univariate findings and highlight pathway-linked metabolites associated with IPF progression and treatment response.

**Fig. 8:**
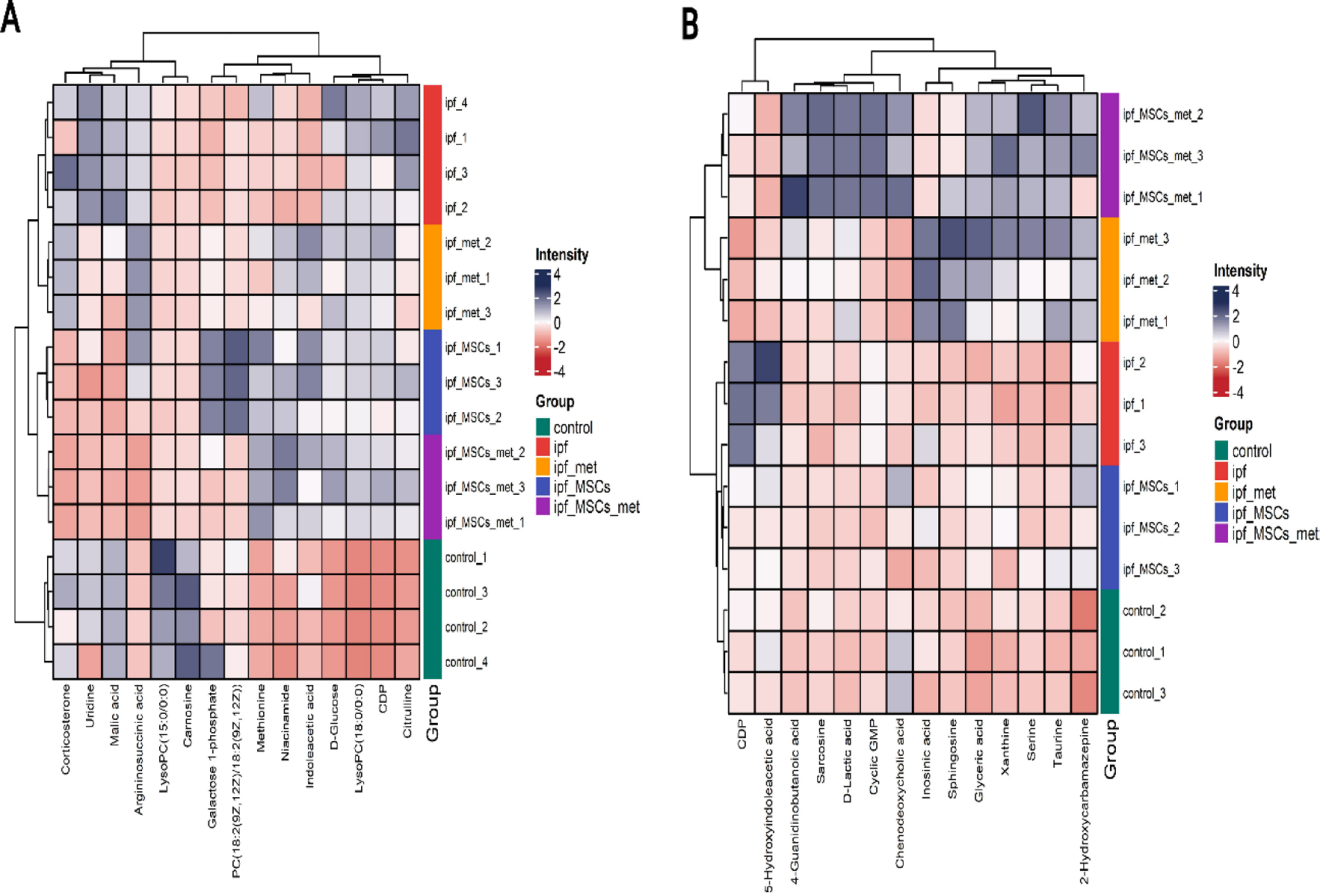
Hierarchical clustering analysis (HCA) heat maps of plasma and lung-tissue differentially expressed metabolites (DEMs). **(A**) Heat map of plasma metabolites. (**B**) Heat map of tissue metabolites.

To complement the PCA, PLS-DA, and VIP analyses, we performed hierarchical clustering analysis (HCA) on the plasma and lung-tissue datasets (Fig. 8). The heat maps visualize sample-level similarity and revealed robust separation of Healthy Control and IPF cohorts, whereas the Metformin-treated, MSC-treated, and Combined Metformin + MSC-treated groups clustered closer to controls. This shift toward control-like profiles in treated animals is consistent with a therapeutic normalization of the metabolome.

## Discussion

### Histopathological corroboration of therapeutic efficacy

Histopathological analysis remains the gold standard for evaluating therapeutic efficacy in preclinical models of idiopathic pulmonary fibrosis (IPF). As expected, bleomycin-induced fibrosis in the IPF group was characterized by extensive interstitial collagen deposition, thickening of alveolar septa, and marked architectural distortion, consistent with previous descriptions of advanced pulmonary fibrosis in rodent models and human disease^3,26^. In contrast, the control group demonstrated normal lung parenchyma with thin alveolar walls and minimal collagen accumulation, thereby validating the model baseline.

Treatment interventions yielded varying degrees of histological improvement. Metformin-treated animals exhibited partial restoration of alveolar architecture and reduced collagen deposition compared to the untreated IPF group. This observation is consistent with prior studies reporting metformin’s antifibrotic activity, which is largely attributed to activation of AMP-activated protein kinase (AMPK) signaling and suppression of TGF-β1–mediated fibroblast activation and extracellular matrix (ECM) production^22,24,25^.

Similarly, mesenchymal stem cell (MSCs) monotherapy attenuated fibrotic remodeling, as evidenced by decreased collagen accumulation and partial reopening of alveolar spaces. This aligns with previous preclinical studies demonstrating that MSCs can migrate to sites of injury, secrete paracrine mediators, modulate inflammation, and promote epithelial repair^25,27,28^.

The most pronounced histological improvement was observed in the combination therapy of (metformin and MSCs), which exhibited nearly normal alveolar architecture with minimal fibrosis. This synergistic effect likely reflects complementary mechanisms: metformin primarily targeting metabolic and AMPK-dependent signaling pathways, while MSCs contribute regenerative and immunomodulatory effects. Previous work in cardiovascular, hepatic, and renal models has suggested that metformin may enhance MSCs survival, engraftment, and paracrine potency within the hostile fibrotic microenvironment^28,30,31^. Collectively, these findings strongly support the superiority of combined therapy over monotherapy in reversing established pulmonary fibrosis and highlight the promise of multi-target therapeutic strategies in IPF^30^.

### Metabolomics signatures reveal systemic impact and therapeutic restoration

Untargeted metabolomics profiling provided a systemic overview of the biochemical perturbations induced by pulmonary fibrosis and the restorative effects of therapeutic interventions. Multivariate analyses, including PCA and PLS-DA (Fig. 7A-D), demonstrated that the IPF group was metabolically distinct from controls, confirming profound systemic metabolic dysregulation associated with fibro genesis^32,36^. Specifically, IPF samples clustered away from controls, reflecting global shifts in carbohydrate, amino acid, lipid, and redox metabolism, which are increasingly recognized as hallmarks of fibrotic remodeling^3,7,26^.

Both metformin and MSCs monotherapies partially shifted the metabolic profile toward that of the control group (Fig. 7A-D), indicating restoration of systemic metabolic balance. However, the combination therapy induced the most pronounced normalization, with clustering patterns positioned closest to controls. This finding suggests that combinatorial therapy exerts a more comprehensive reprogramming of metabolism than monotherapy alone. Such convergence toward healthy profiles has been reported in prior metabolomics studies of antifibrotic interventions, where restoration of homeostasis rather than complete reversal of individual metabolites is predictive of therapeutic benefit^37,38^.

Hierarchical clustering analysis (HCA; Fig. 8A-B) further supported these observations, revealing distinct segregation of control and IPF groups, while treated groups exhibited closer alignment with controls. Notably, the combined therapy of metformin and MSCs demonstrated the most cohesive clustering pattern, underscoring its superior efficacy in harmonizing systemic metabolic signatures. These findings are consistent with recent systems biology studies suggesting that fibrosis is sustained by interconnected metabolic circuits, and effective therapy requires coordinated correction across multiple pathways^39,40^.

Taken together, these metabolomics results parallel with the histopathological findings (Fig. 3), highlighting the capacity of metformin and MSCs—particularly in combination—to counteract the global metabolic dysregulation underlying fibrotic progression. This integrated histological and metabolomics evidence strongly supports the rationale for multi-target therapeutic strategies in IPF.

Consistent with the separation observed in multivariate analyses (Figs. 7 and 8), classification of metabolites by main chemical classes demonstrated that both plasma and tissue metabolomes were dominated by carboxylic acids and derivatives, followed by lipid-related metabolites (Fig. 2). Carboxylic acids encompass amino acids and their derivatives, which play a central role in fibroblast activation, extracellular matrix synthesis, and redox homeostasis in IPF^41^. Lipid-related metabolites, particularly glycerophospholipids and sphingolipids, are increasingly recognized as drivers of fibroblast proliferation and profibrotic signaling through pathways such as lysophosphatidic acid and sphingosine-1-phosphate^42,43^. The predominance of these classes in both systemic (plasma) and local (lung-tissue) profiles suggests that metabolic dysregulation in IPF extends beyond the lung microenvironment, reflecting systemic remodeling. These observations provide a biochemical framework for the more specific metabolite-level and pathway-level alterations identified in the univariate analyses (Figs. 4 and 6).

### Mechanistic insights from Key metabolite alterations

In addition to the global metabolic shifts captured by PCA, PLS-DA, and HCA (Figs. 7,8), univariate analyses identified nine metabolites that were significantly altered across the experimental cohorts (Fig. 4). These metabolites represent critical biochemical nodes spanning carbohydrate, amino acid, lipid, nucleotide, and neuroendocrine metabolism. Their consistent modulation in response to therapy highlights not only their role in the pathogenesis of pulmonary fibrosis but also their utility as potential biomarkers of treatment efficacy. As shown in Fig. 6 mapping these alterations onto Kyoto Encyclopedia of Genes and Genomes (KEGG) pathways revealed specific hubs of dysregulation, including galactose metabolism (map00052), methionine metabolism (map00270), arginine and creatine metabolism (map00330/map00260), nicotinate and NAD metabolism (map00760), glycerophospholipid metabolism (map00564), sphingolipid metabolism (map00600), steroid biosynthesis (map00140), tryptophan metabolism (map00380), and cGMP–PKG signaling (map04022). As represented in Figs. 4,5,6 Galactose 1-phosphate, mapping to galactose metabolism (map00052), was markedly decreased in the IPF group relative to all treatment groups, consistent with impaired carbohydrate flux and reduced entry into UDP-sugar biosynthesis, which is essential for collagen glycosylation and proper extracellular matrix assembly^44^. Restoration of Gal-1-P in treated groups suggests normalization of ECM turnover, aligning with reports that disordered carbohydrate metabolism fuels fibrogenesis in lung tissue^45^. Methionine (map00270) was significantly elevated in IPF compared to healthy control, MSCs-treated, and the combined therapy of Metformin and MSCs, reflecting dysregulation of methylation balance and redox homeostasis. Given its role as the precursor of S-adenosylmethionine, a universal methyl donor and glutathione substrate, excess methionine points to disrupted one-carbon metabolism and oxidative stress activation in IPF^46^. The return to baseline with MSCs monotheapy is consistent with stem cell–induced redox stabilization and epigenetic reprogramming of fibroblasts. Niacinamide (plasma; map00760) was higher in IPF than the Metformin-treated and the combination therapy, pointing to altered NAD⁺/NADH homeostasis and mitochondrial energetics. Its reduction by metformin-containing arms aligns with AMPK activation and improved redox resilience^47,48^. Corticosterone (map00140), a key glucocorticoid hormone, was elevated in IPF relative to the MSCs-treated and the combined therapy, consistent with maladaptive hypothalamic–pituitary–adrenal axis activation in chronic injury^49^. The capacity of MSCs therapy to reduce corticosterone reflects systemic immunomodulation and endocrine stabilization, mechanisms that may attenuate pro-fibrotic inflammation. PC (18:2(9Z, 12Z)/18:2(9Z, 12Z)), a glycerophospholipid (map00564), was significantly higher in IPF compared to the MSCs-treated, aligning with evidence that phosphatidylcholine dysregulation enhances autotaxin-mediated lysophosphatidic acid (LPA) signaling and fibroblast activation^45^. Its correction with MSCs implies diversion of phospholipid metabolism away from the pro-fibrotic LPA axis. Sphingosine, within the sphingolipid pathway (map00600), was uniquely altered in the Metformin-treated group compared to IPF and healthy controls, indicating that Metformin specifically modulates ceramide–sphingosine–S1P signaling. Since S1P promotes fibroblast proliferation and resistance to apoptosis^50^, this metformin-induced shift may reflect suppression of pro-survival lipid cues. 

5-Hydroxyindoleacetic acid (5-HIAA), the principal serotonin metabolite (map00380), was elevated in the MSCs-treated compared to IPF and healthy controls, pointing to enhanced serotonin turnover. Given serotonin’s established role in stimulating fibroblast proliferation and ECM deposition^51^^,52^, this finding suggests that MSCs may accelerate serotonin clearance and thereby blunt pro-fibrotic signaling. Cyclic GMP (map04022) was significantly increased in the MSCs-treated and the combined therapy groups compared to IPF, supporting activation of the cGMP–PKG pathway, a well-described antifibrotic axis that antagonizes TGF-β signaling and suppresses myofibroblast differentiation^53^. Finally, 4-guanidinobutanoic acid, linked to arginine and creatine metabolism (map00330/map00260), was uniquely elevated in the combined therapy group, suggesting synergistic restoration of nitrogen and energy buffering systems. As creatine metabolism intersects with nitric oxide production^54^, its modulation may reduce fibroblast contractility and normalize vascular signaling.

Beyond the nine significantly altered metabolites, two unique glycolytic intermediates were identified, namely dihydroxyacetone phosphate (DHAP) and fructose-1, 6-bisphosphate (F1, 6BP). Elevated DHAP in the IPF group is consistent with enhanced glycolytic flux and diversion into lipid biosynthesis, a metabolic phenotype increasingly recognized in activated fibroblasts. Conversely, the reduction of F1, 6BP in IPF relative to healthy controls and MSCs-treated groups points to impaired activity of phosphofructokinase, a key regulatory step in glycolysis^55^. Similar decreases in F1, 6BP have been reported in both experimental and clinical metabolomics studies of pulmonary fibrosis, linking attenuated glycolytic capacity with fibrotic remodeling^17^. The partial restoration of F1, 6BP in the MSCs-treated animals suggests that stem cell therapy may normalize glycolytic balance, aligning with previous evidence of metabolic correction following antifibrotic interventions^17^. Together, the identification of DHAP and F1, 6BP underscores the central role of glycolytic dysregulation in IPF and nominates glycolysis as a therapeutic target. More broadly, the metabolite signatures indicate that IPF is maintained by interconnected disturbances in carbohydrate flux, one-carbon/methylation and redox balance, phospholipid and sphingolipid signaling, neuroendocrine stress responses, serotonin turnover, nucleotide signaling, and nitrogen metabolism. While Metformin and MSCs treatments each corrected distinct subsets of these pathways, the combined therapy achieved the broadest and most integrative metabolic restoration, corroborating histological evidence of synergy and underscoring the therapeutic necessity of multi-axis interventions in pulmonary fibrosis.

### Contextual comparison with FDA-approved antifibrotics

The current standard of care for idiopathic pulmonary fibrosis is FDA-approved antifibrotic drugs like pirfenidone and nintedanib, but their therapeutic benefit is mainly restricted to slowing the disease’s progression rather than reversing fibrosis that has already developed^56,57^. Pirfenidone has been demonstrated to reduce collagen deposition, suppress transforming growth factor-β signaling, and partially improve lung mechanics in experimental bleomycin-induced pulmonary fibrosis models, whereas nintedanib primarily inhibits multiple tyrosine kinase pathways involved in fibroblast migration and proliferation to produce its antifibrotic effects^58,59^. Nevertheless, neither medication completely restores normal lung architecture, nor after treatment, residual fibrotic lesions usually remain.

In this regard, the degree of antifibrotic reactions seen in this study after the administration of metformin and mesenchymal stem cells is comparable to the effects of nintedanib and pirfenidone in comparable bleomycin-based models. According to earlier research, pirfenidone and nintedanib reduce lung hydroxyproline content and histological fibrosis scores by about 30–50%. They have little effect on metabolic remodeling and only minor effects on epithelial regeneration^58,59^. On the other hand, our results show that metformin and mesenchymal stem cells, especially when taken together, have more extensive antifibrotic effects that go beyond the suppression of extracellular matrix. These effects include modulation of metabolic dysregulation, reduction of oxidative stress, and improvement of tissue repair mechanisms.

By inducing myofibroblast dedifferentiation through AMP-activated protein kinase–dependent metabolic reprogramming—a pathway not directly targeted by traditional antifibrotic medications—metformin has been demonstrated to mechanistically promote fibrosis resolution^60^. Similarly, by improving alveolar epithelial repair, reducing profibrotic immune responses, and reestablishing redox homeostasis in fibrotic lungs, mesenchymal stem cells support antifibrotic effects^61^. These mechanisms are essentially complementary to those of nintedanib and pirfenidone, which mainly work by suppressing fibroblast activity and profibrotic signaling instead of encouraging active tissue regeneration.

Taken together, preclinical data indicates that the therapeutic effects seen in this study are at least comparable to those reported for pirfenidone and nintedanib in bleomycin-induced pulmonary fibrosis models, even though direct head-to-head comparisons with FDA-approved antifibrotic agents were not possible within the current experimental design. Significantly, the multimodal effects of metformin and mesenchymal stem cells point to their potential use as supplemental or alternative therapeutic approaches to improve antifibrotic efficacy and target pathogenic pathways that are not adequately addressed by existing pharmacological treatments.

## Synthesis and implications

The integration of histological and metabolomic findings provides a comprehensive understanding of the therapeutic potential of combined metformin and MSCs therapy in IPF. Both structural (histological) and functional (metabolomic) assessments demonstrate that while either treatment alone confers partial benefit, their combination elicits a more profound and coordinated reversal of fibrosis. Restoration of lung tissue architecture was paralleled by the normalization of systemic metabolic pathways, underscoring the interplay between local fibrotic remodeling and whole-body metabolic homeostasis.

## Limitations

Despite the promising outcomes demonstrated in this study, several limitations should be acknowledged. First, the experimental model relied exclusively on bleomycin-induced pulmonary fibrosis in rats, despite, it is well-established, does not fully capture the chronic and heterogeneous nature of human idiopathic pulmonary fibrosis (IPF). Second, the sample size, though adequate for a pilot study, limits the statistical power and generalizability of the findings. Third, the use of untargeted metabolomics provides a broad overview of metabolic changes but lacks quantitative precision and pathway-specific resolution. Future work employing targeted metabolomics could better validate and quantify key metabolic biomarkers. Additionally, the study focused on a single time point post-treatment; thus, the long-term effects and stability of the observed metabolic restoration remain unknown. Finally, the absence of functional assays or molecular pathway validations (e.g., Western blot or RT-qPCR for TGF-β or AMPK signaling) limits mechanistic interpretation.

Building on these findings, future research should pursue longitudinal studies in larger animal cohorts to evaluate the durability and reproducibility of the observed therapeutic effects. Translational studies using human-derived samples or ex vivo IPF models will be critical to confirm the clinical relevance of the identified metabolic alterations.

Integration of multi-omics platforms—including transcriptomics, proteomics, and lipidomics—will provide a more comprehensive understanding of how metabolic, inflammatory, and fibrotic pathways converge in IPF. The development of targeted metabolomics assays for these biomarkers would facilitate their translation into clinical monitoring tools. In parallel, mechanistic studies should focus on unraveling the molecular crosstalk between metabolic and fibrotic signaling networks, particularly those involving cGMP–PKG signaling, sphingolipid metabolism, and serotonin turnover.

From a therapeutic standpoint, further work is needed to optimize MSC delivery strategies (including dosing, frequency, and route of administration) and to explore rational combination regimens with other antifibrotic agents. Such approaches may enhance efficacy and accelerate clinical translation, ultimately paving the way toward personalized, multi-targeted therapies capable of halting or reversing the progression of IPF.

## Future perspectives and clinical translation

In experimental pulmonary fibrosis, the present study provides preclinical evidence supporting the synergistic anti-fibrotic potential of combined mesenchymal stem cell (MSCs) and metformin therapy. From a clinical translation perspective, metformin’s well-established safety profile and oral route of administration represent clear advantages for rapid clinical implementation. In parallel, MSCs can be delivered intravenously or intratracheally, both of which have already been explored in early-phase clinical trials for interstitial lung diseases. Future studies should focus on optimizing MSCs dosing, timing, and delivery routes, as well as evaluating advanced strategies such as MSCs preconditioning and the therapeutic application of MSCs-derived extracellular vesicles to enhance efficacy and reproducibility. Moreover, the metabolic biomarkers identified in this study warrant further validation as non-invasive indicators of disease progression and therapeutic response, which could facilitate patient stratification and longitudinal treatment monitoring in future clinical trials. Collectively, these findings highlight the translational promise of combined MSCs–metformin therapy as a novel, multi-targeted therapeutic strategy for idiopathic pulmonary fibrosis and provide a rational framework for its advancement toward clinical evaluation.

## Conclusion

This study provides compelling evidence that idiopathic pulmonary fibrosis (IPF) is characterized not only by profound histopathological alterations but also by extensive metabolic dysregulation. The integration of histological findings with multivariate and targeted metabolomic analyses revealed that while metformin and MSCs individually confer partial benefits, their combination achieves a more complete reversal of fibrotic pathology. Restoration of alveolar architecture was consistently mirrored by normalization of systemic metabolic signatures.

Nine metabolites—Galactose-1-phosphate, Methionine, Niacinamide, Corticosterone, 4-Guanidinobutanoic acid, Sphingosine, 5-HIAA, Cyclic GMP, and PC(18:2/18:2)—emerged as critical markers of disease activity and therapeutic response. Their modulation highlighted key pathways including carbohydrate metabolism, one-carbon and redox balance, NAD⁺ metabolism, amino acid turnover, lipid signaling, neurotransmitter degradation, and antifibrotic cGMP–PKG signaling. Importantly, the combination therapy restored balance across these interconnected networks, underscoring the need for multi-axis therapeutic strategies in IPF.

These findings not only deepen our understanding of the metabolic underpinnings of fibrosis but also identify a panel of candidate biomarkers with translational potential for disease monitoring and treatment evaluation. By simultaneously correcting metabolic dysfunction with metformin and enhancing regenerative and immunomodulatory mechanisms with MSCs, the combination therapy achieved a synergistic effect that exceeded the benefits of either agent alone.

Overall, this work highlights the promise of metabolomics-guided interventions in IPF and establishes a foundation for future translational studies aimed at validating these biomarkers and advancing combination therapies toward clinical application.

## Supplementary Information

Below is the link to the electronic supplementary material.

Supplementary Material 1

Supplementary Material 2

Supplementary Material 3

Supplementary Material 4

## Data Availability

Processed metabolomics data and metadata are provided as supplementary material with this article, which are sufficient for independent reanalysis and reproducibility of the results. All the codes used for data analysis and graphical visualization are available in the GitLab repository under project ID 73,719,878 (https://gitlab.com/prolab11/idiopathic-pulmonary-fibrosis). In addition, the data that support the findings of this study are openly available in the EMBL-EBI MetaboLights database. The raw metabolomics data can be accessed through the following accession number: MTBLS12893 (https://www.ebi.ac.uk/metabolights/MTBLS12893).
