# Supplementary material for: Combined mesenchymal stem cells and metformin therapy modulates key macromolecular pathways in pulmonary fibrosis based on evidence from untargeted metabolomics
Source: Sci Rep. 2026 Apr 24;16:14641. doi: 10.1038/s41598-026-46691-8 (PMC13153430; doi:10.1038/s41598-026-46691-8)
Supplement: Supplementary file 4 — Supplementary Material 4 [file 41598_2026_46691_MOESM4_ESM.pdf]

This document contains supplementary figures and tables supporting the main manuscript. Supplementary figures and Tables 1–3 are provided within the Supplementary PDF file, while Supplementary Tables 4–6 are provided as separate Excel files

Table No.	Title / Description	Location / File
Supplementary Table 1	Changes in body weight between different groups during both induction and therapeutic phase	Supplementary PDF
Supplementary Table 2	A list of the relatively quantified metabolites and their sample types and adjusted p-value.	Supplementary PDF
Supplementary Table 3	Markers of Oxidative Stress and Tissue Remodeling Across Study Groups	Supplementary PDF
Supplementary Table 4	Full metabolites list	Excel File 1 (.csv)
Supplementary Table 5	Positive metabolites group	Excel File 2 (.csv)
Supplementary Table 6	Negative metabolites group	Excel File 3 (.csv)

Figure No.	Title / Description	File Format
Supplementary Fig. 1	Changes in body weight between different groups during both induction and therapeutic phase	PDF
Supplementary Fig. 2	Work flow of sample preparation for metabolomics analysis.	PDF
Supplementary Fig. 3	UHPLC-MS/MS analytical workflow	PDF
Supplementary Fig. 4	Lung histology – control group (MTC stain)	PDF
Supplementary Fig. 5	Lung histology – IPF group (MTC stain)	PDF
Supplementary Fig. 6	Lung histology – Metformin treated group (MTC stain)	PDF
Supplementary Fig. 7	Lung histology – MSCs treated group (MTC stain)	PDF
Supplementary Fig. 8	Lung histology – Combination treated group (MTC stain)	PDF
Supplementary Fig. 9	Univariate analysis of plasma and lung-tissue metabolomics profiles	PDF
Supplementary fig.10	Heat map (Plasma origin)	PDF
Supplementary fig.11	Heat map (Tissue origin)	PDF

Table S.1. Shows changes in body weight between different groups during both induction and therapeutic phase

Day/Phase	Negative control (SD)	IPF (SD)	Met (SD)	MSCs (SD)	Combined (SD)
0 (Induction phase)	200 ± 10	198 ± 9	–	–	–
10 (Induction phase)	215 ± 11	213 ± 10	–	–	–
20 (Induction phase)	245 ± 13	238 ± 12	–	–	–
30 (Induction phase)	272 ± 14	262 ± 13	–	–	–
40 (Induction phase	280 ± 16	267 ± 15	–	–	–
50 (Induction phase)	288 ± 18	272 ± 17	272 ± 17	272 ± 17	272 ± 17
60 (Therapeutic phase)	320 ± 22	285 ± 20	290 ± 21	315 ± 24	293 ± 21
70 (Therapeutic phase)	332 ± 24	287 ± 21	294 ± 22	323 ± 26	304 ± 23
80 (Therapeutic phase)	340 ± 26	289 ± 22	296 ± 23	331 ± 27	314 ± 24
90 (Therapeutic phase)	350 ± 28	292 ± 23	312 ± 25	340 ± 28	322 ± 26

Table S.2. A list of the relatively quantified metabolites and their sample types and adjusted p-value.

METABOLITE NAME	HMDB ID	P. ADJUSTED	SAMPLE TYPE
**GLYCERIC ACID**	HMDB0000139	0.0106	Tissue
**INOSINIC ACID**	HMDB0000175	3.95×10^{-3}	Tissue
**SERINE**	HMDB0000187	0.026052	Tissue
**TAURINE**	HMDB0000251	0.0054276	Tissue
**SPHINGOSINE**	HMDB0000252	0.0010715	Tissue
**SARCOSINE**	HMDB0000271	2.91×10^{-5}	Tissue
**XANTHINE**	HMDB0000292	0.035618	Tissue
**CHENODEOXYCHOLIC ACID**	HMDB0000518	0.037588	Tissue
**5-HYDROXYINDOLEACETIC ACID**	HMDB0000763	0.031121	Tissue
**D-LACTIC ACID**	HMDB0001311	1.5151×10^{-6}	Tissue
**CYCLIC GMP**	HMDB0001314	4.7284×10^{-7}	Tissue
**CDP**	HMDB0001546	1.5151×10^{-6}	Tissue
**4-GUANIDINOBUTANOIC ACID**	HMDB0003464	0.0039484	Tissue
**2-HYDROXYCARBAMAZEPINE**	HMDB0060651	0.043082	Tissue
**CARONSINE**	HMDB0000033	3.12×10^{-5}	Plasma
**ARGINOSUCCINIC ACID**	HMDB0000052	0.00016782	Plasma
**D-GLUCOSE**	HMDB0000122	0.01574	Plasma
**MALIC ACID**	HMDB0000156	1.99×10^{-5}	Plasma
**INDOLEAACETIC ACID**	HMDB0000197	0.014548	Plasma
**URIDINE**	HMDB0000296	0.0028639	Plasma
**GALACTOSE 1- PHOSPHATE**	HMDB0000645	0.014182	Plasma
**METHIONINE**	HMDB0000696	0.0010181	Plasma
**CITRULLINE**	HMDB0000904	0.00089239	Plasma
**NIACINAMIDE**	HMDB0001406	0.0021625	Plasma
**CDP**	HMDB0001546	0.00045367	Plasma
**CORTICOSTERONE**	HMDB0001547	0.012127	Plasma
**PC(18:2(9Z,12Z)/18:2(9Z,12Z))**	HMDB0008138	1.9×10^{-7}	Plasma
**LYSOPC(15:0/0:0)**	HMDB0010381	0.00013799	Plasma
**LYSOPC(18:0/0:0)**	HMDB0010384	3.84×10^{7}	Plasma

Table S.3. Markers of Oxidative Stress and Tissue Remodeling Across Study Groups

Phase	Group	MDA (nmol/mg tissue)	MMP-9 (ng/g tissue)	Serum Albumin (g/dL)
Induction	Healthy Control (HC)	150 ± 12.3	25 ± 3.9	3.2 ± 0.2
Induction	Idiopathic pulmonary fibrosis (IPF)	200 ± 15.6	32 ± 4.8	2.6 ± 0.2
Therapeutic	Healthy Control (HC)	150 ± 13.4	26 ± 3.6	3.3 ± 0.2
Therapeutic	Idiopathic pulmonary fibrosis untreated (IPF)	260 ± 18.7	37 ± 5.3	2.4 ± 0.2
Therapeutic	Metformin-treated (Met)	280 ± 20.8	34 ± 4.2	2.8 ± 0.3
Therapeutic	Mesenchymal stem cells (MSCs)-treated	230 ± 17.3	29 ± 4.6	2.6 ± 0.2
Therapeutic	Metformin + MSCs-treated	140 ± 11.5	26 ± 3.7	2.9 ± 0.2

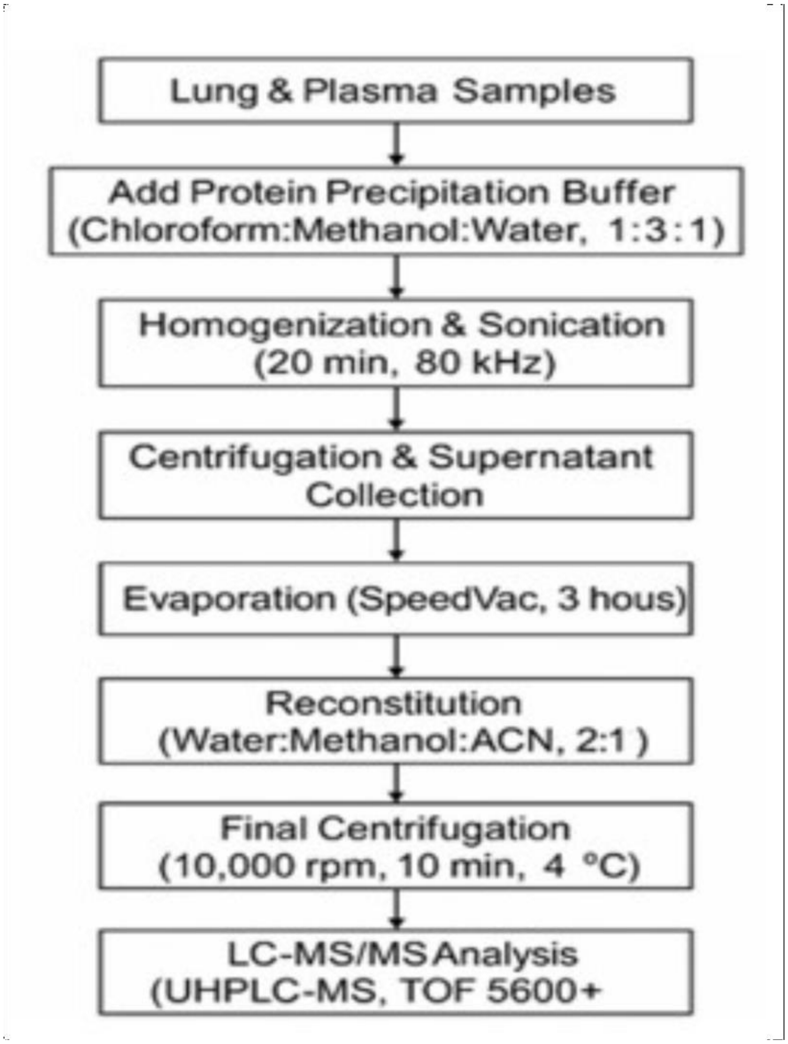

Figure S.1. Work flow of sample preparation for metabolomics analysis.

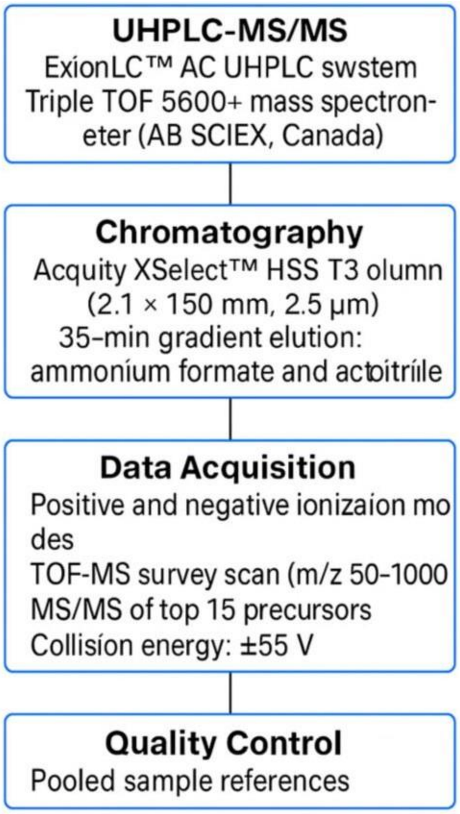

Figure.S.2.UHPLC-MS/MS analytical workflow.

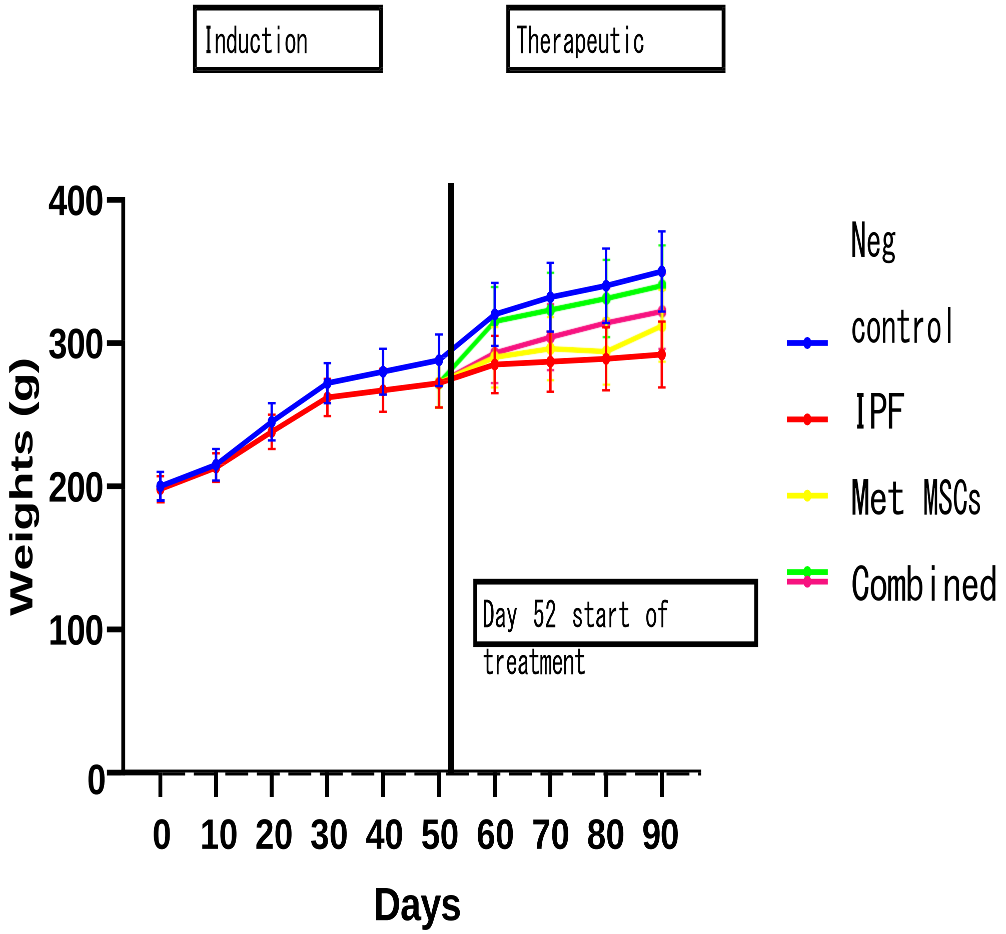

Figure S.3. Shows changes in body weight between different groups during both induction and therapeutic phase

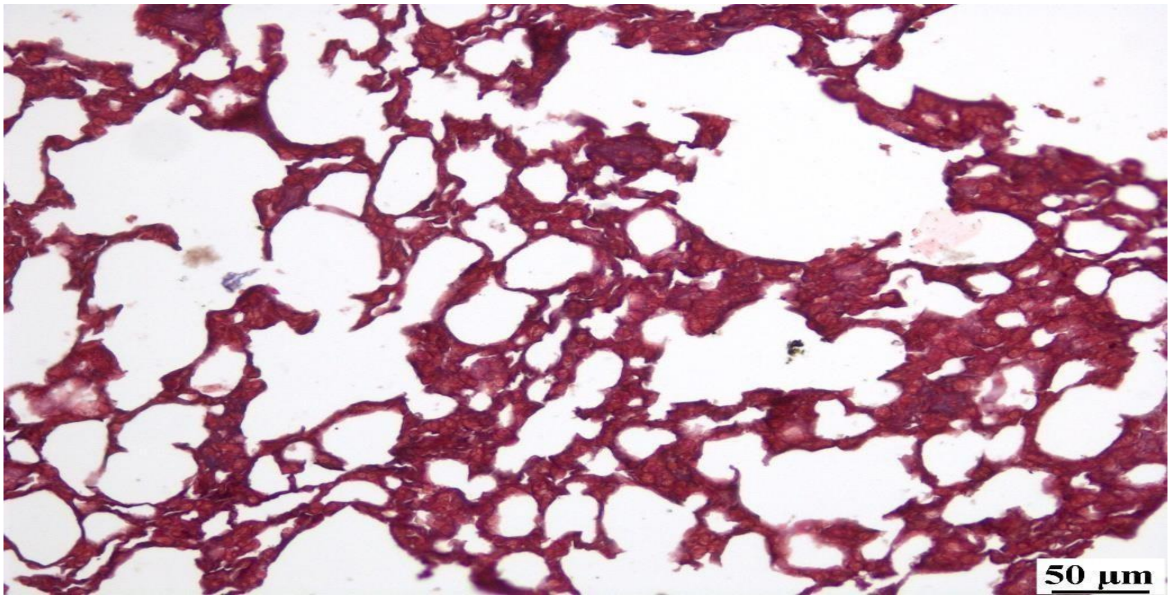

Figure S.4. Lung histology – control group (MTC stain):

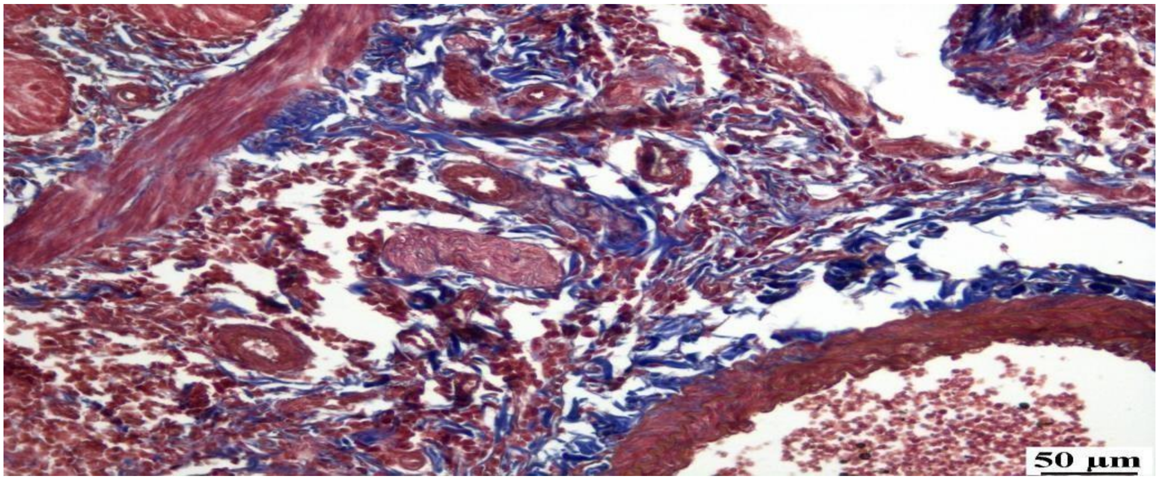

Figure S.5. Lung histology – IPF positive control group (MTC stain):

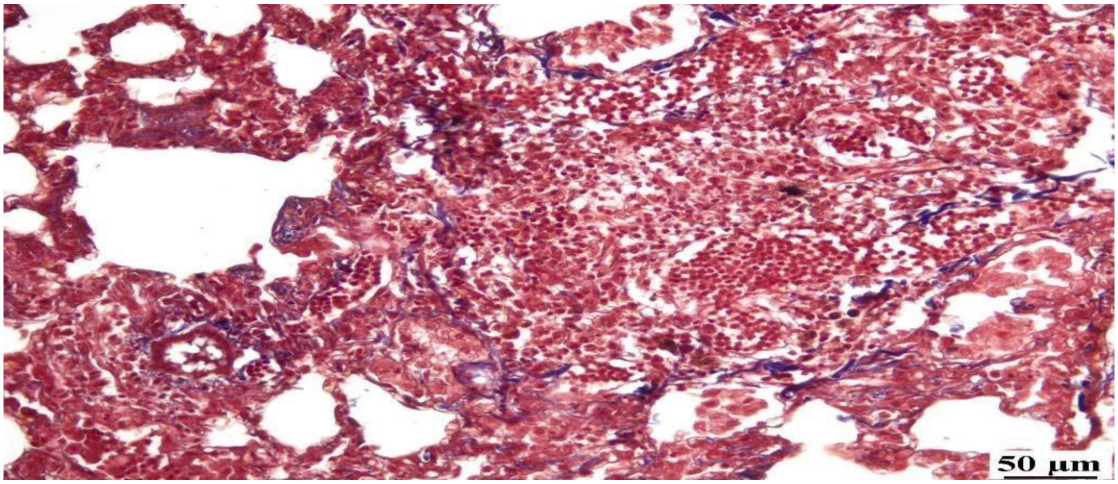

Figure S.6. Lung histology – metformin treatment group (MTC stain)

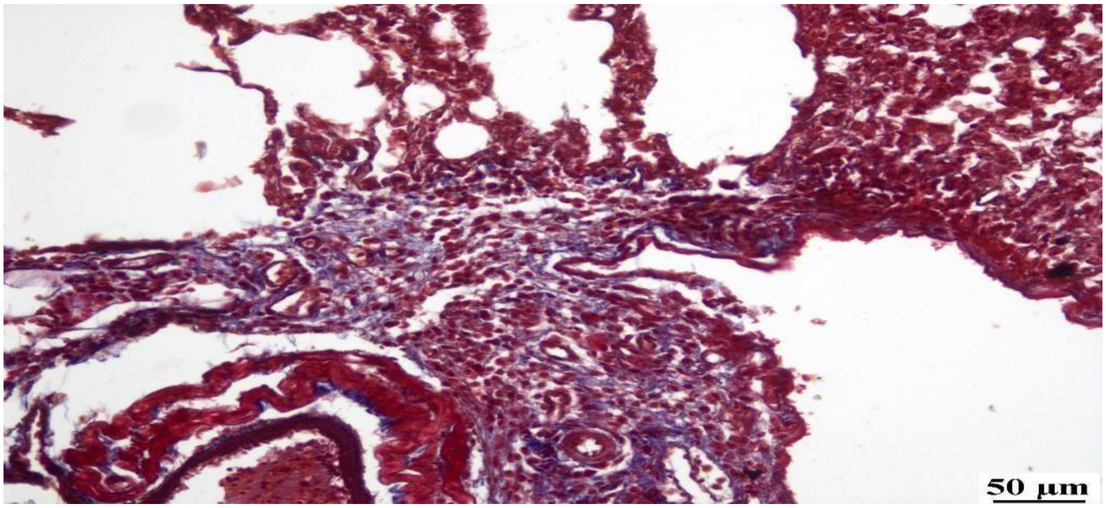

Figure S.7. Lung histology – MSC treatment group (MTC stain)

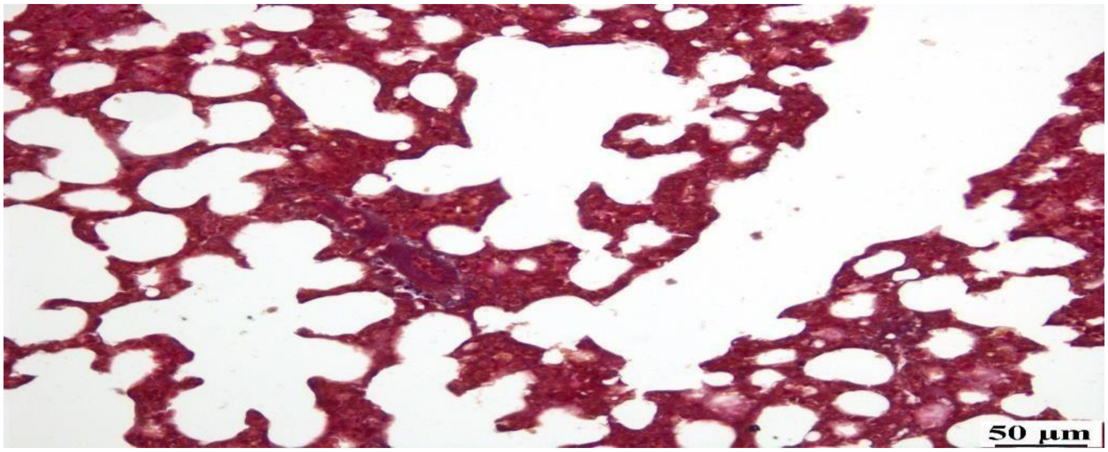

FigureS.8. Lung histology – combination therapy group (MTC stain)

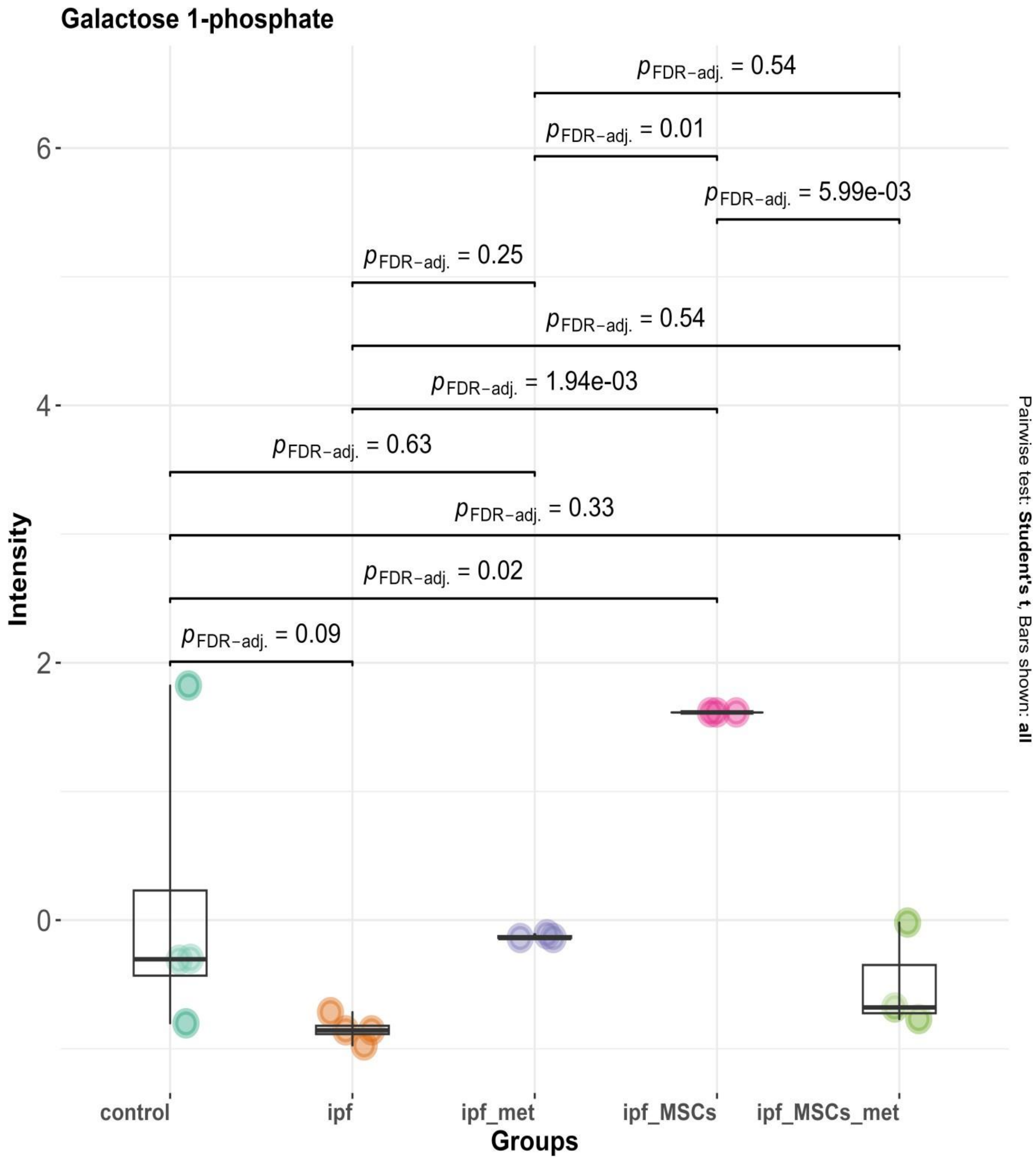

Figure S.9A. Galactose-1-phosphate box plot.

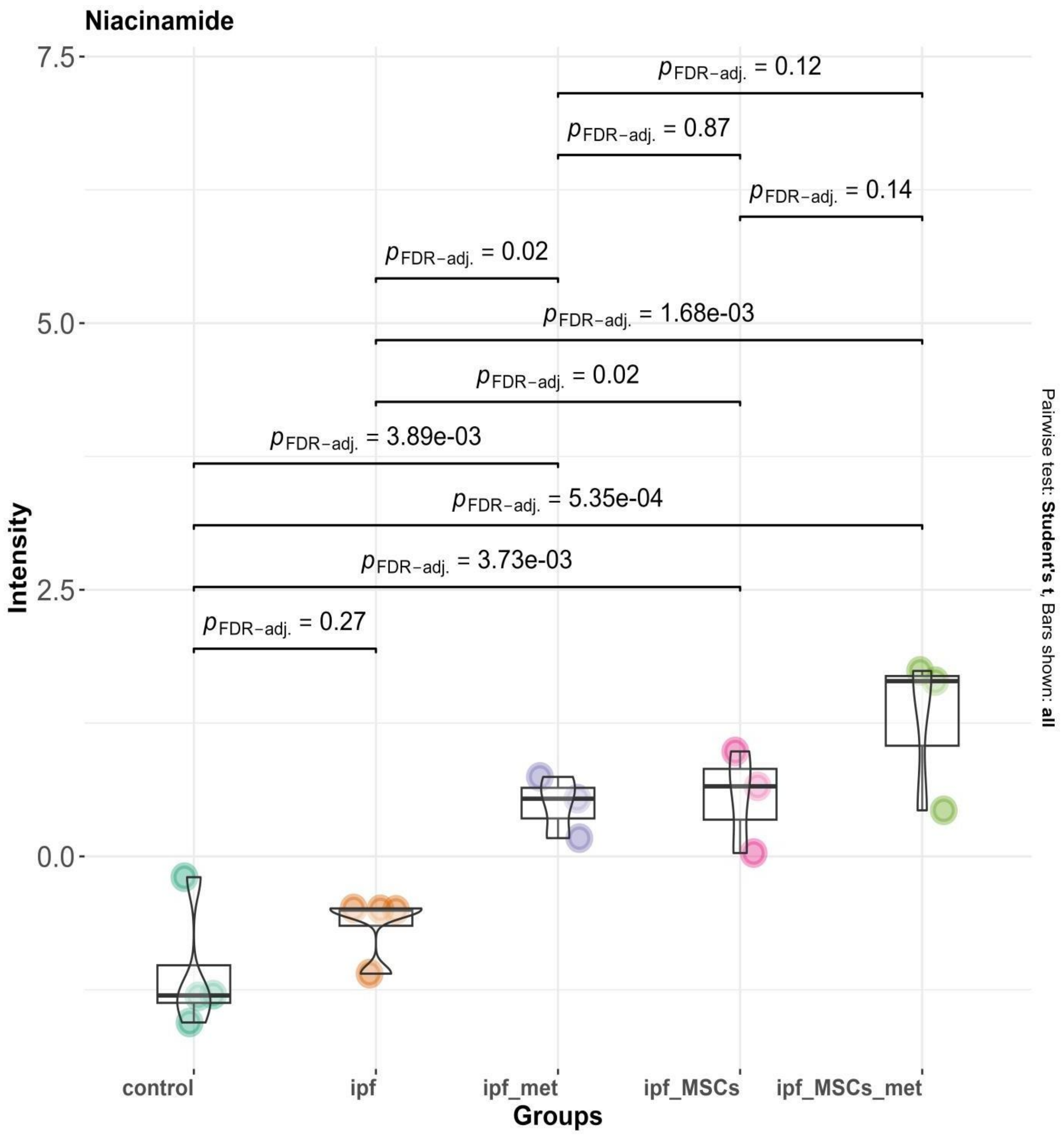

Figure S.9B. Niacinamide box plot.

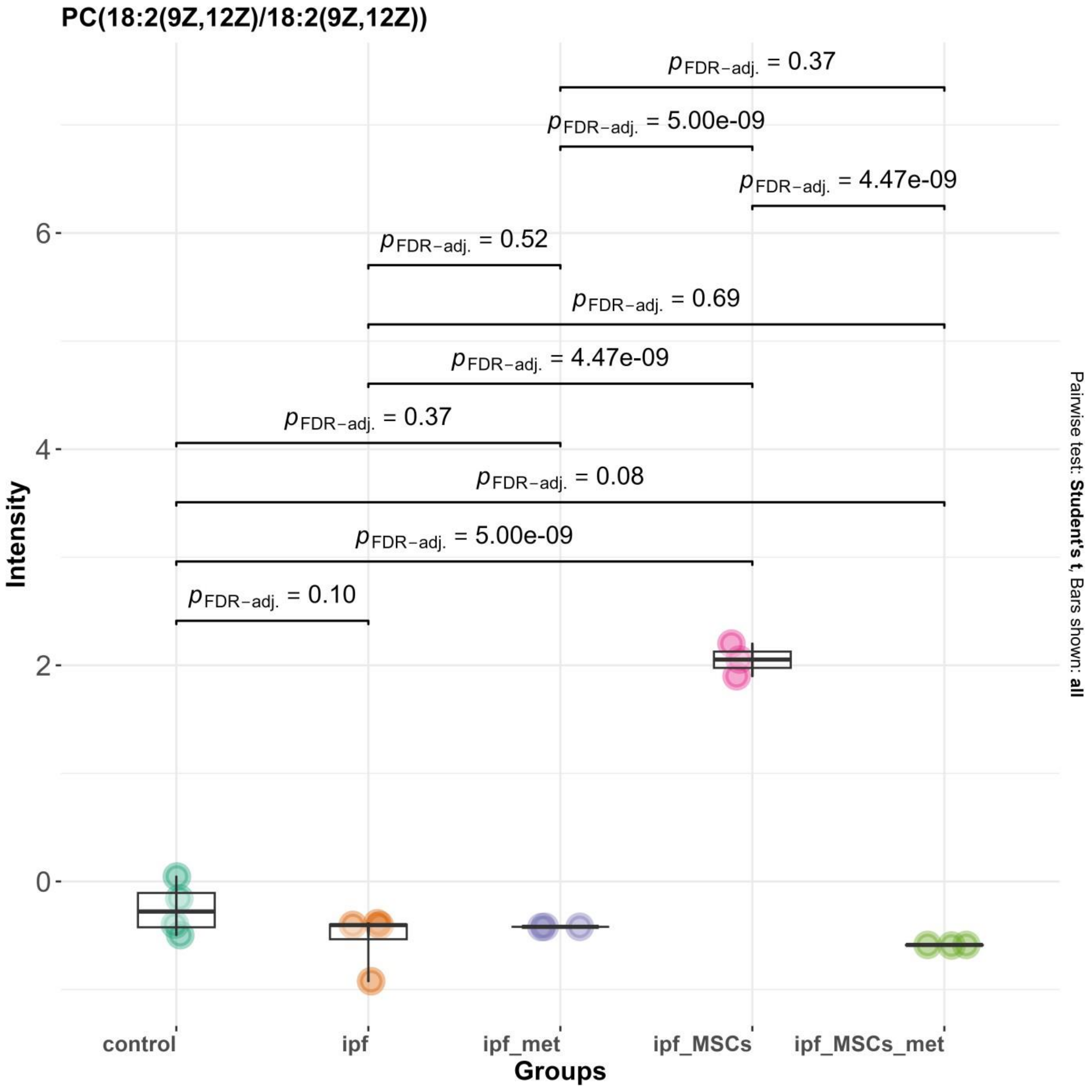

Figure S.9C. PC (18:2(9Z, 12Z)/18:2(9Z, 12Z)) boxplot.

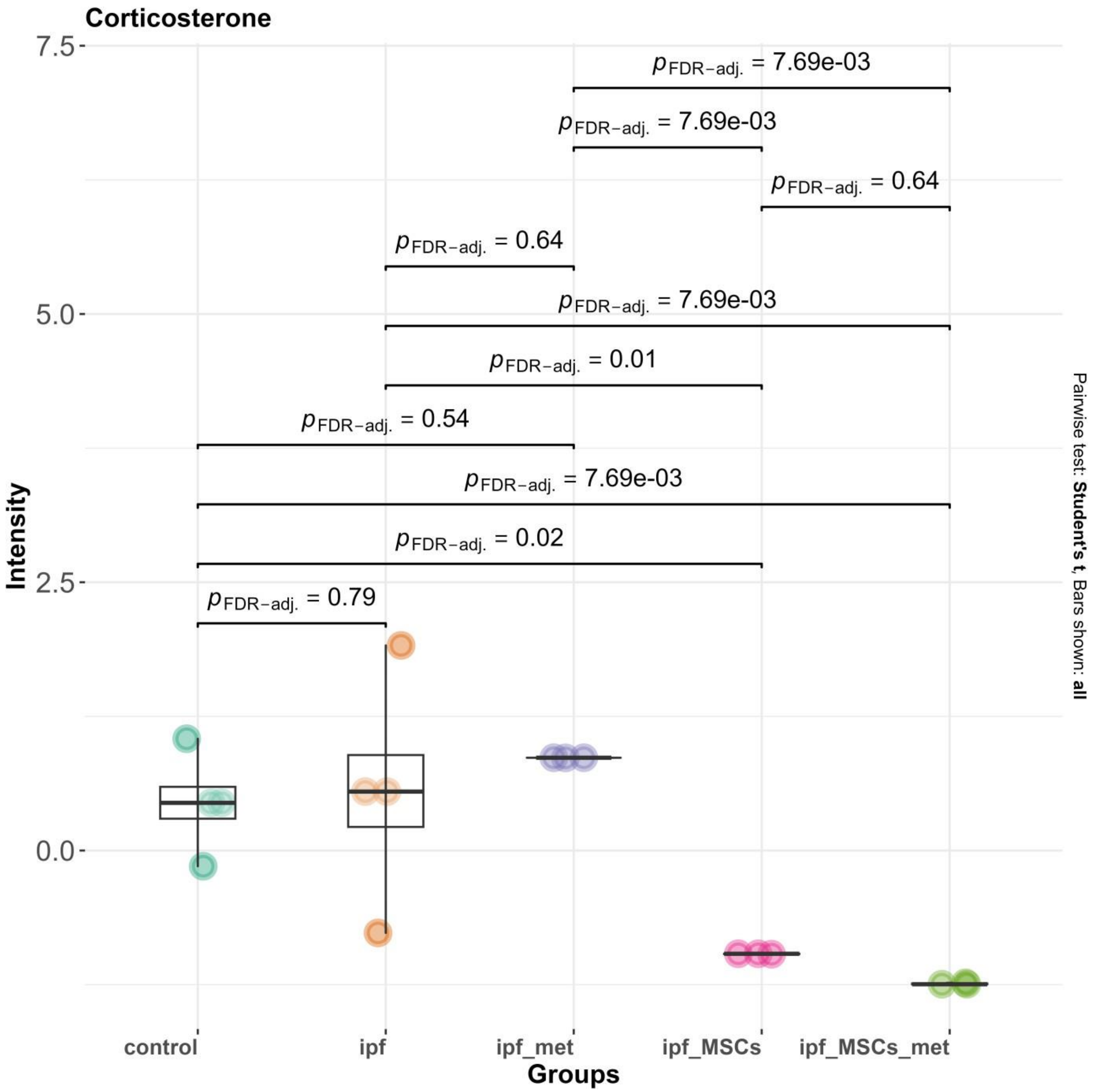

Figure S.9D. Corticosterone boxplot.

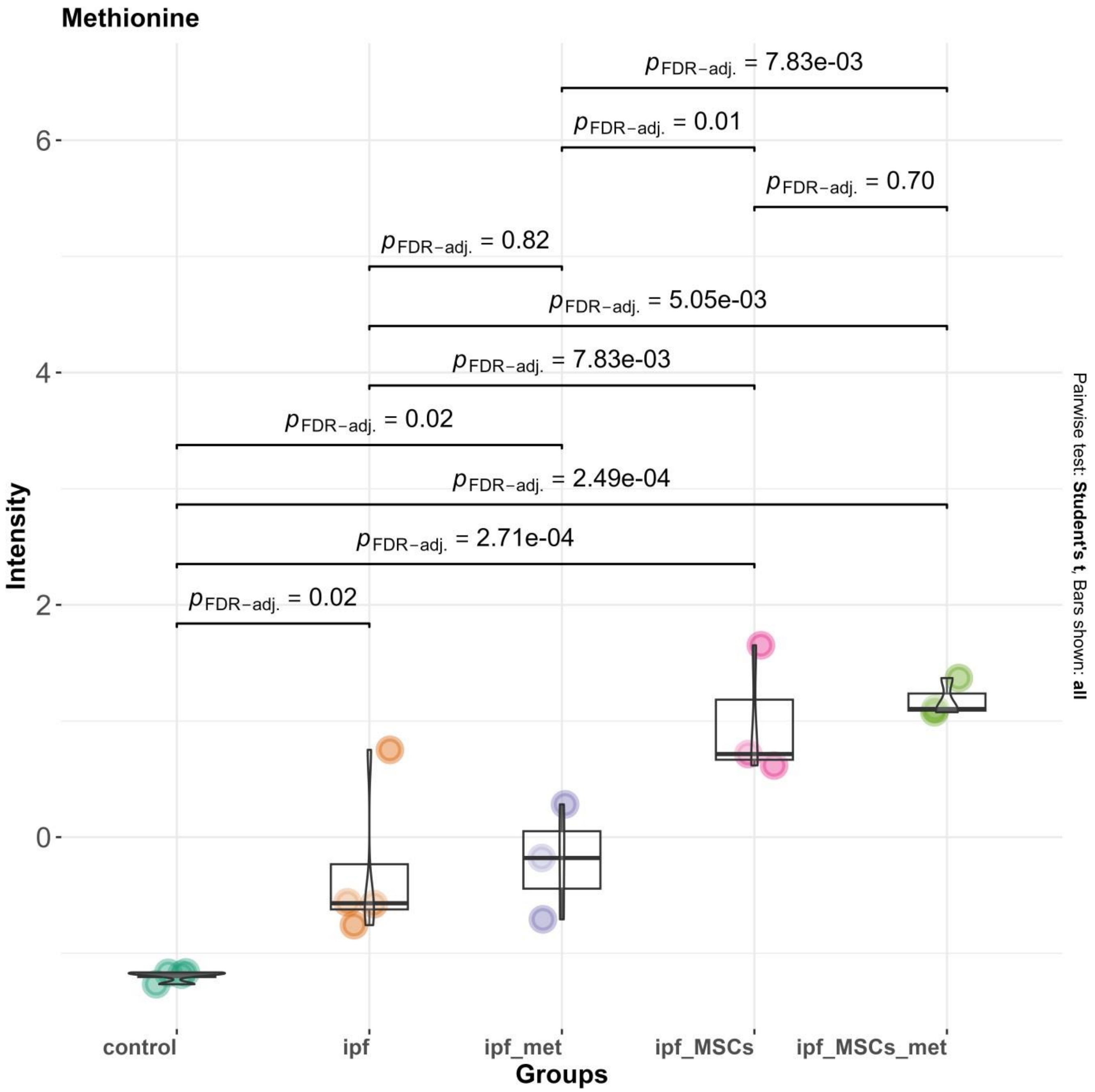

Figure S.9E. Methionine boxplot.

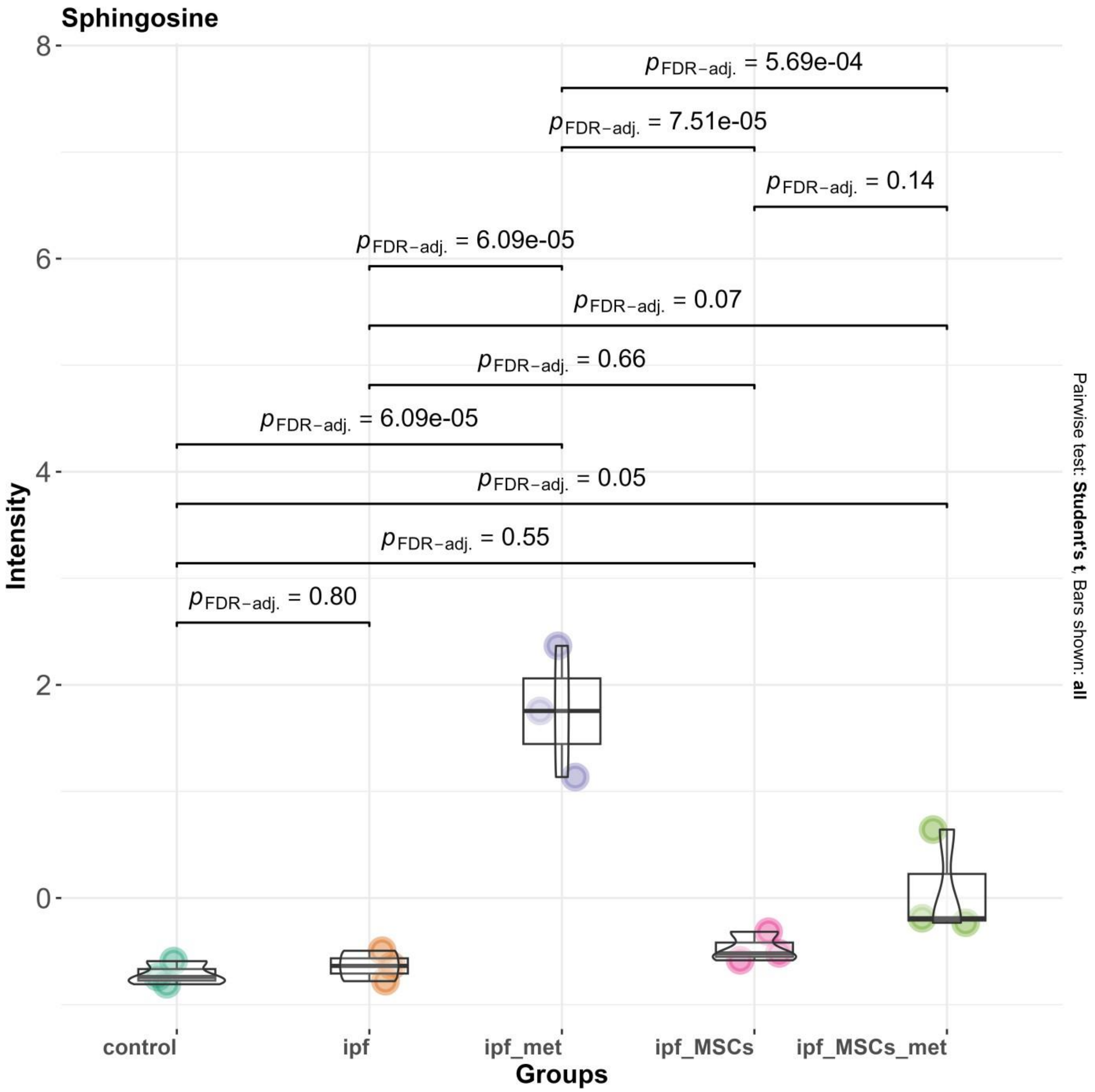

Figure S.9F. Sphingosine boxplot.

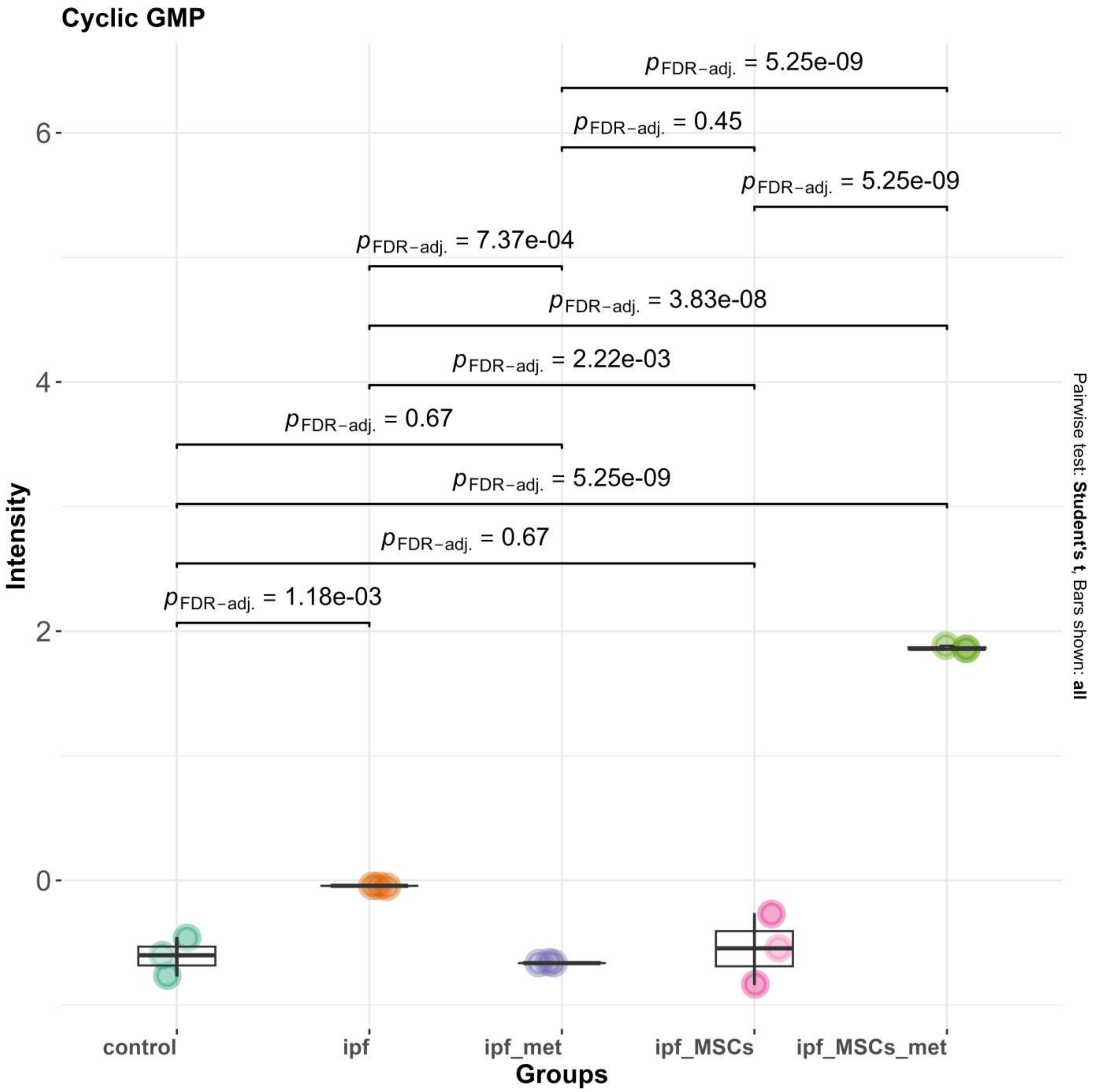

Figure S.9G. Cyclic GMP boxplot.

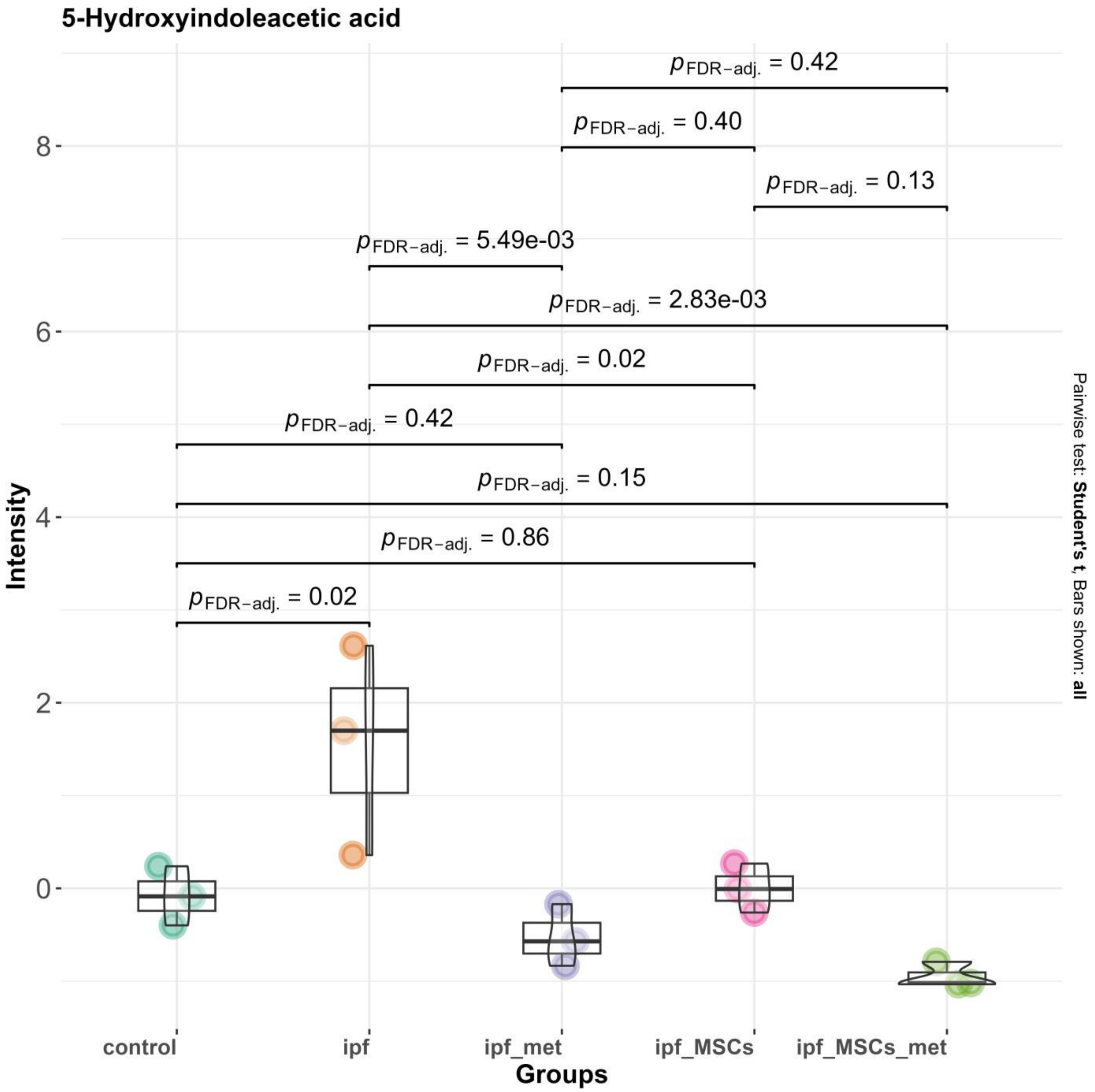

Figure S.9H. 5-Hydroxyindoleacetic acid boxplot.

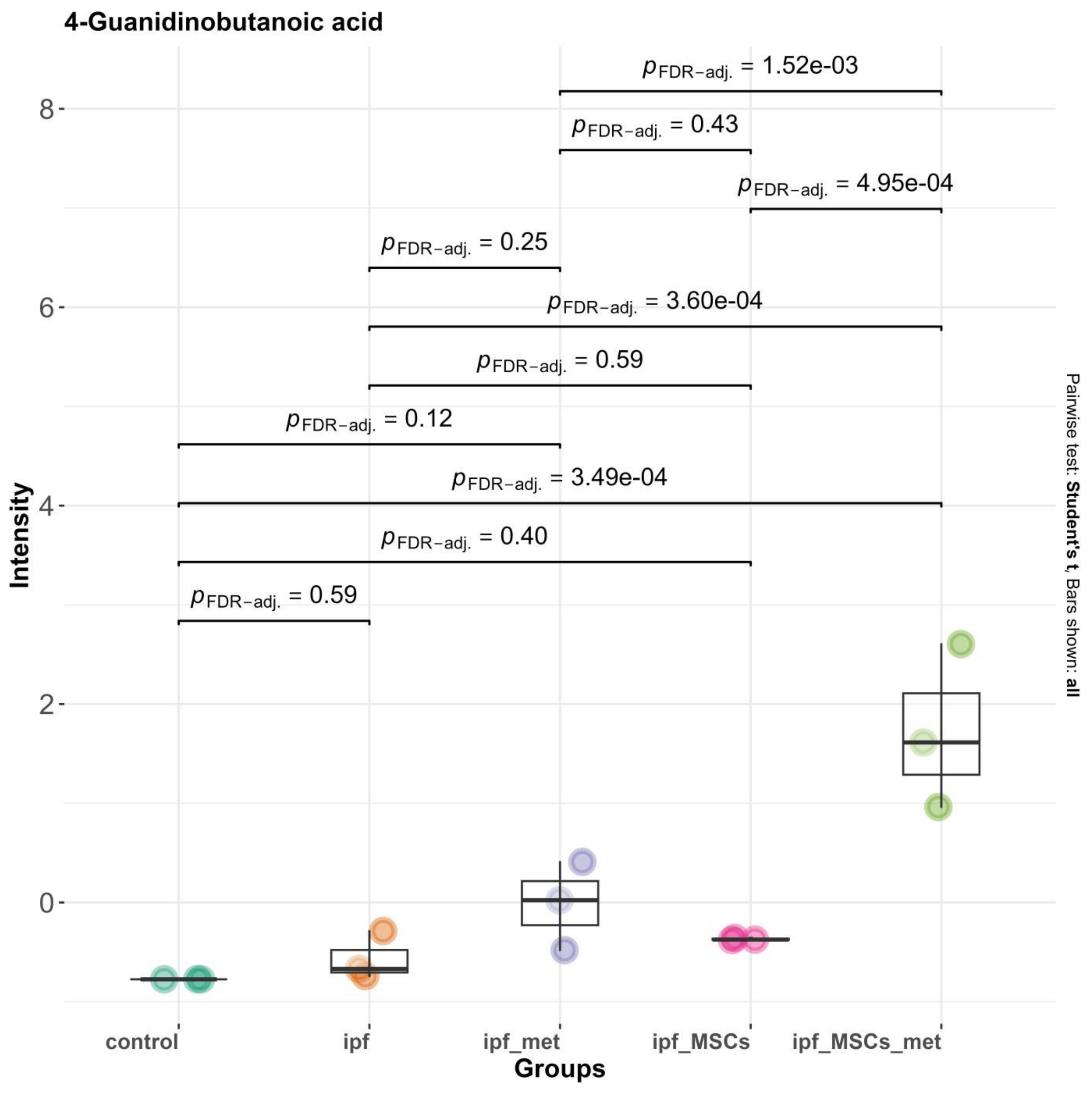

Figure S.9I. 4-Guanidibutanoic acid boxplot.

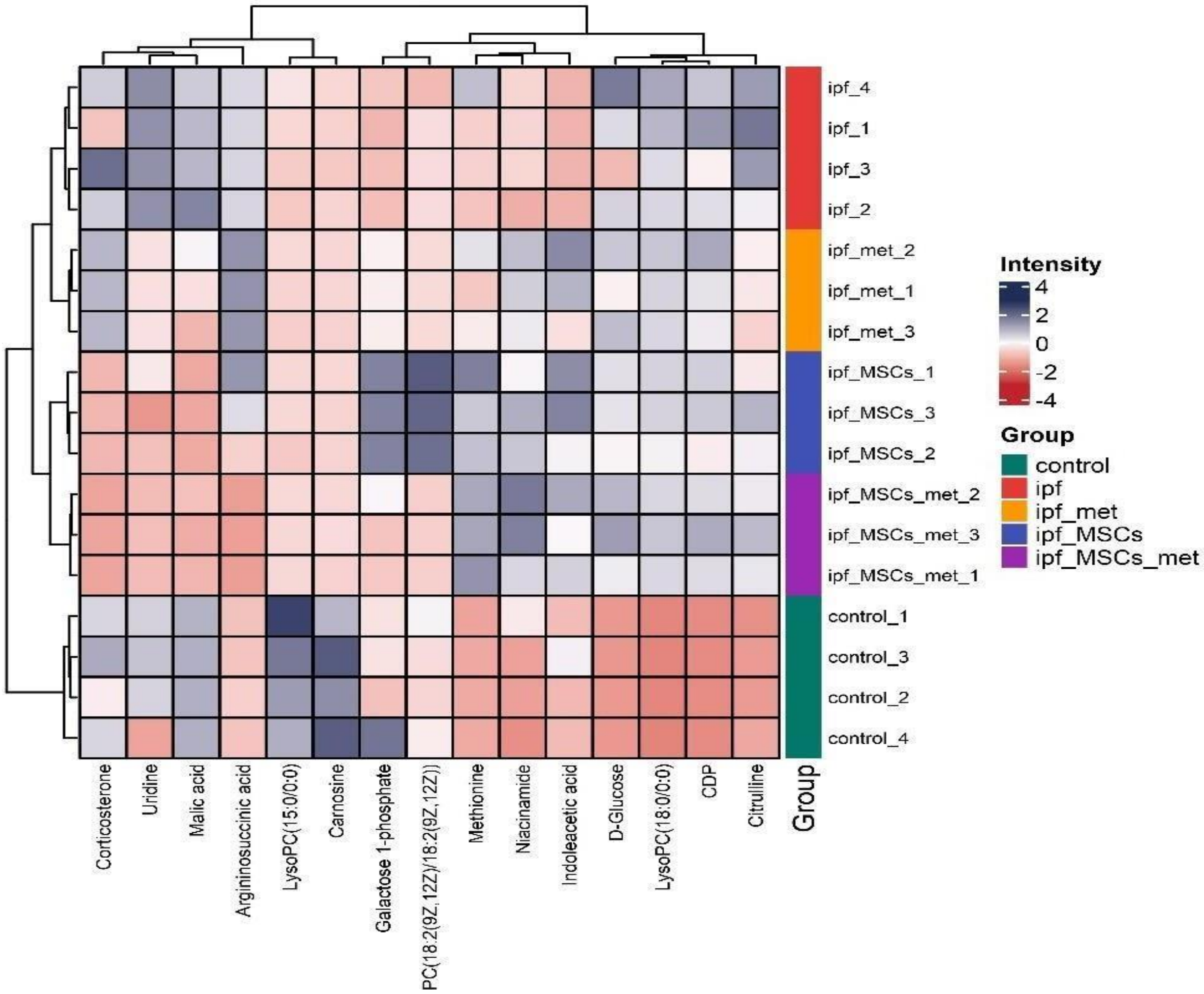

Figure S.10. Heat map (Plasma origin).

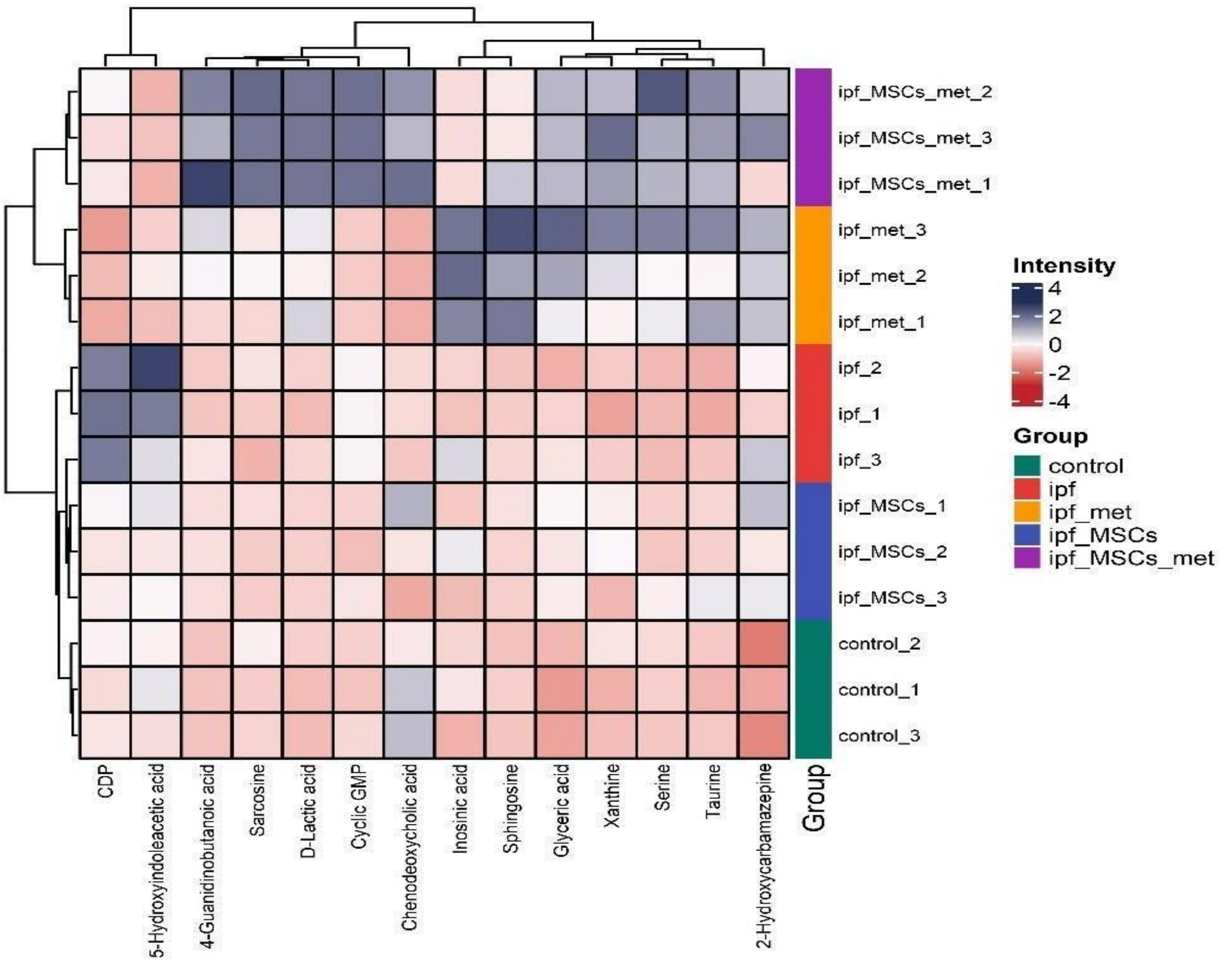

Figure S.11. Heat map (Tissue origin
